# AIM2 deficiency in B cells ameliorates systemic lupus erythematosus by regulating Blimp-1–Bcl-6 axis-mediated B-cell differentiation

**DOI:** 10.1038/s41392-021-00725-x

**Published:** 2021-09-14

**Authors:** Ming Yang, Di Long, Longyuan Hu, Zhidan Zhao, Qianwen Li, Yunkai Guo, Zhenghao He, Ming Zhao, Liwei Lu, Fen Li, Hai Long, Haijing Wu, Qianjin Lu

**Affiliations:** 1 Department of Dermatology, Second Xiangya Hospital, Central South University, Hunan Key Laboratory of Medical Epigenomics, Changsha, Hunan China; 2 Department of Otolaryngology Head and Neck Surgery, Second Xiangya Hospital, Central South University, Changsha, China; 3 Department of Pathology and Shenzhen Institute of Research and Innovation, The University of Hong Kong, Pok Fu Lam, Hong Kong; 4 Department of Rheumatology and Immunology, Second Xiangya Hospital, Central South University, Changsha, China; 5 Institute of Dermatology, Chinese Academy of Medical Sciences and Peking Union Medical College, Beijing, China

**Keywords:** Rheumatic diseases, Immunological disorders

## Abstract

Absent in melanoma 2 (AIM2) has been reported to be a component of inflammasomes in innate immune cells. Surprisingly, AIM2 is expressed by B cells, and higher AIM2 expression is observed in the B cells from lupus patients. To date, the inflammasome-independent function of AIM2 in B cells remains unclear. Here, we report increased expression of AIM2 in human tonsil memory and germinal center (GC) B cells and in memory B cells and plasma cells from the circulation and skin lesions of lupus patients. Conditional knockout of AIM2 in B cells reduces the CD19^+^ B-cell frequency in lymph nodes and spleens, and dampens KLH-induced IgG1-antibody production. In a pristane-induced mouse model of lupus, AIM2 deficiency in B cells attenuates lupus symptoms and reduces the frequency of GC B cells, T follicular helper (Tfh) cells, plasmablast cells, and plasma cells. Furthermore, the loss of AIM2 in human B cells leads to the increased expression of Blimp-1 and reduces the expression of Bcl-6. However, the silencing of Blimp-1 and Bcl-6 has no significant effect on AIM2 expression, indicating that AIM2 might be the upstream regulator for Blimp-1 and Bcl-6. In addition, IL-10 is found to upregulate AIM2 expression via DNA demethylation. Together, our findings reveal that AIM2 is highly expressed in the B cells of lupus patients and promotes B-cell differentiation by modulating the Bcl-6–Blimp-1 axis, providing a novel target for SLE treatment.

## Introduction

Absent in melanoma 2 (AIM2) is originally identified as a tumor suppressor of melanoma and named absent in melanoma 2.^1^ AIM2 is a member of the interferon (IFN)-inducible PYHIN protein family.^2^ In immune cells, AIM2 is mainly observed in innate immune cells, such as macrophages and dendritic cells, and functions to sense pathogen-associated or host-derived cytosolic dsDNA, recruit other inflammasome components, such as ASC and pro-caspase-1, and induce caspase-dependent inflammasome formation. The AIM2-inflammasome can further promote either IL-18 and IL-1β production or gasdermin-D (GSDMD)-mediated pyroptosis.^3–6^ In addition, the inflammasome-independent function of AIM2 is also linked to the regulation of intestinal cell proliferation, apoptosis, and metastasis in colon cancer.^7^ However, whether AIM2 exerts inflammasome-independent effects in immune cells, especially in adaptive immune cells, is unclear.

Systemic lupus erythematosus (SLE) is an autoimmune disease characterized by abundant autoantibody production.^8^ Therefore, B cells, which are the main source of antibodies, are believed to be the dominant immune cells that contribute to the immune abnormalities observed in SLE. In the peripheral circulation, mature B cells are activated by self- or foreign antigens and then differentiate into either memory B cells or antibody-producing plasma cells.^9^ After germinal center (GC) responses, B cells ultimately differentiate into plasma cells. Although memory B cells are not capable of secreting antibodies, they can further undergo somatic hypermutation (SHM) and/or class switch DNA recombination (CSR) and then differentiate into plasma cells upon subsequent antigen exposure.^10^ This process is mainly regulated by the B lymphocyte-induced maturation protein 1 (Blimp-1)-B-cell lymphoma 6 protein (Bcl-6) axis.

Blimp-1 (encoded by *Prdm1*) controls the differentiation of plasma cells. Bcl-6 is well documented as the key transcription factor for GC B-cell differentiation. Prior to differentiation, Blimp-1 is suppressed by Bcl-6. It has been reported that the increased expression of *Prdm1* may result from the release of Bcl-6-bound histone deacetylases (HDACs), thereby increasing the histone acetylation levels in the promoter region of *Prdm1.*^11^ Blimp-1 represses the gene transcription of *Bcl6*, *Pax5*, and *Spib*, which, in turn, inhibits Blimp-1 transcription and GC B and plasma cell differentiation.^12^ Thus, the Bcl-6-Blimp-1 axis controls B-cell differentiation.

Here, we first described an increased level of AIM2 in human tonsil memory and GC B cells and in memory B cells and plasma cells from the peripheral blood from lupus patients. To further explore the function of AIM2 in B cells, we constructed CD19 conditional knockout AIM2 mice. The loss of AIM2 in B cells reduced KLH-induced antibody production and ameliorated lupus symptoms. Mechanistically, IL-10 was found to upregulate AIM2 expression via DNA demethylation, and AIM2 was found to directly bind to Blimp-1 and Bcl-6, as well as regulate their expression. Our findings may provide a novel target for lupus therapy.

## Results

### Increased AIM2 expression in B-cell subtypes from SLE patients

**Fig. 1:**
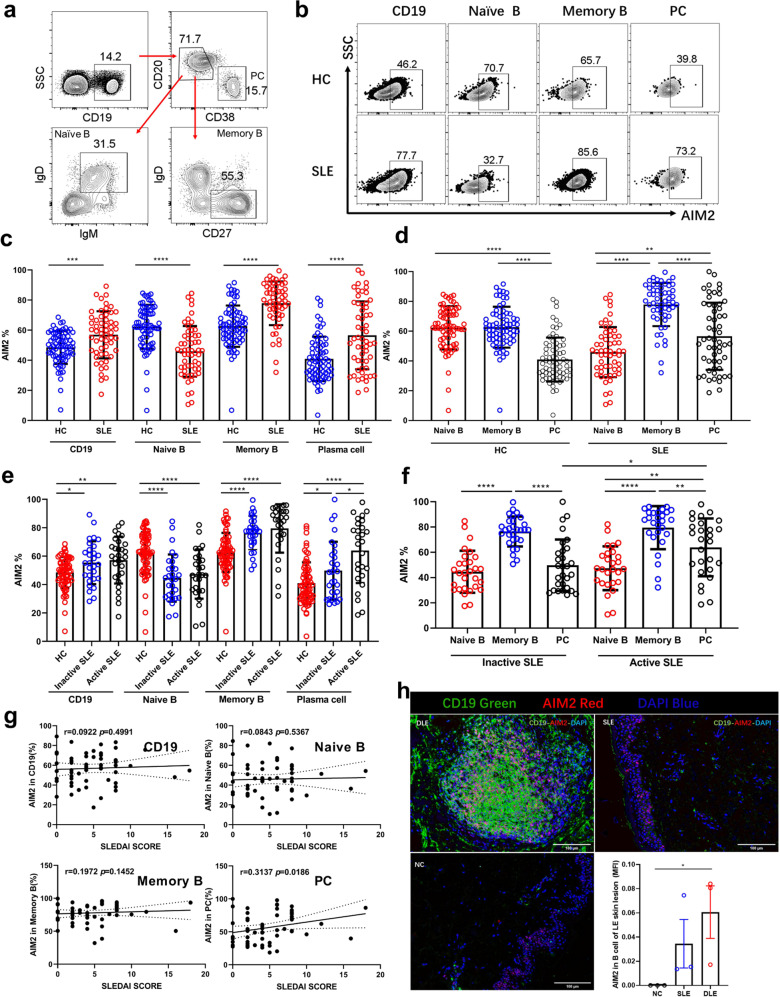
The expression of AIM2 in circulating B cells and skin B cells from SLE patients. The expression of AIM2 was detected by flow cytometry and multicolor microscopy. **a** Representative flow diagram of gating for plasma cells, naive B cells, and memory B cells. **b** Representative flow diagram of AIM2 expression in plasma cells, naive B cells, and memory B cells from HC subjects (*n* = 70), inactive SLE patients (*n* = 29), and active SLE patients (*n* = 27). **c**–**g** Statistical analysis of AIM2^+^ cells in populations of plasma cells, naive B cells, and memory B cells. **h** The location and expression levels of AIM2 in normal control (NC), SLE, and DLE skin samples. CD19^+^ B cells are in green, and AIM2^+^ cells are in red. Horizontal bars represent the mean ± SEM. *p < 0.05, **p < 0.01, ***p < 0.005, ****p < 0.0001

To further investigate whether the AIM2 expression is associated with disease conditions, we detected the AIM2 expression in different B-cell subtypes in the peripheral blood from SLE patients (*n* = 56). As shown in Supplementary Fig. 1, increased numbers of CD19^+^ B, naive B, memory B, and plasma cells were observed in SLE patients. Compared with healthy controls (HCs) (*n* = 70), CD19^+^ B cells, memory B cells and plasma cells from SLE patients showed higher expression of AIM2, except for naive B cells (Fig. 1a–c). Among all these cell types, memory B cells showed the highest AIM2 expression (Fig. 1d). Active SLE patients showed higher expression of AIM2 in plasma cells (Fig. 1e, f), compared with inactive SLE patients. Furthermore, the frequency of AIM2^+^ plasma cells was positively correlated with the SLE disease activity index (SLEDAI) (*r* = 0.3137, *p* = 0.0186, Fig. 1g).

Meanwhile, as shown in Supplementary Fig. 1c, other components of inflammasome were also observed. The mRNA levels of Nlrp3, Nlrp12, and Nlrc4 were significantly increased in SLE B cells, but no significant difference was found in Asc, Nlrp1, Nlrp6, and lfi16 (Supplementary Fig. 1c). To exclude the pharmacological effects on AIM2 expression, Dexamethasone (Dex) was utilized to treat B cells. As shown in Supplementary Fig. 1d, Dex can significantly reduce the mRNA expression of AIM2, Asc, Nlrp1, Nlrp3, Nlrp6, and Nlrp12, but no significant effect on the expression of Ifi16 and Nlrc4. This result indicates that the enhanced expression of AIM2 in SLE B cells is not a result of steroid treatments.

As expected, AIM2 expression was increased in the dermis of skin lesions from discoid lupus erythematosus (DLE) patients compared with that from SLE patients and normal control (NC) subjects (Fig. 1h, *n* = 3). More CD19^+^ B cells infiltrated the dermis of DLE patients, and these B cells formed B-cell clusters, which might respond to local antigens and undergo SHM in situ. Fewer B cells and AIM2^+^ cells were observed in SLE skin lesions, and none of these AIM2^+^ cells were B cells. In another unpublished T cell study, we confirmed that these AIM2^+^ cells were CD4^+^ T cells.

To assess the expression of AIM2 in B cells, we first detected the expression of AIM2 in different subtypes of B cells from human tonsils. As shown in Supplementary Fig. 2a-c, compared with naive B cells, memory B cells and GC B cells displayed higher protein levels of AIM2 (*n* = 21) in the frequency of AIM2^+^ cells. Furthermore, the expression of Blimp-1 and Bcl-6 was also observed in different subtypes of tonsil B cells (Supplementary Fig. 2d, e). Lower expression of Bcl-6 was found in plasma cells (Supplementary Fig. 2e).

To further explore the location and expression of AIM2 in tonsil samples, multiple-colored staining was performed. As shown in Supplementary Fig. 2f, a positive population of AIM2^+^ cells (in green) surrounded CD19^+^ B cells and CD4^+^ T cells in the follicles. Interestingly, Bcl-6^hi^ B cells showed relatively lower expression of AIM2, indicating that AIM2 might regulate B-cell differentiation and be related to the expression of Bcl-6.

### Reduced B cell and increased T cell frequency were found in CD19^cre^AIM2^f/f^ mice

To further explore the function of AIM2 in B cells, we constructed CD19^cre^AIM2^f/f^ mice (Supplementary Fig. 3a), in which AIM2 was conditionally knocked out in CD19^+^ B cells (Supplementary Fig. 3b). Consistent with our previous findings, AIM2 was expressed in the nucleus of B cells but not in the cytoplasm (Supplementary Fig. 3c).

**Fig. 2:**
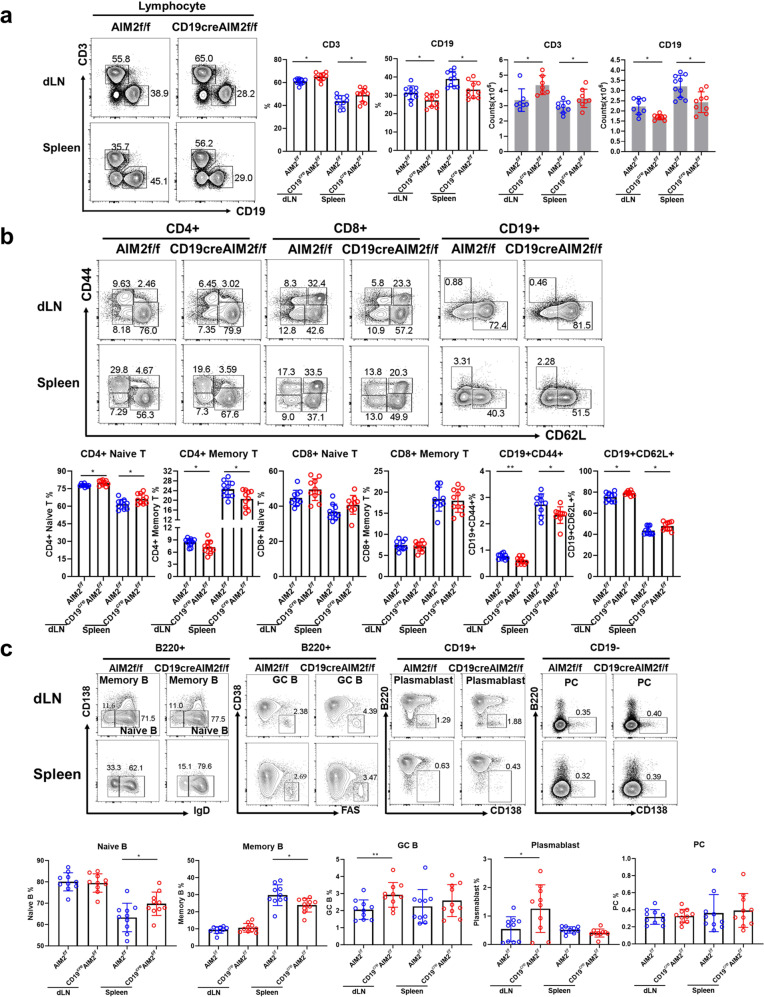
The phenotype of CD19^cre^AIM2^f/f^ mice. The expression of cell-specific surface markers was detected by flow cytometry. **a** Representative flow diagrams and statistical analysis of the percentages of CD3^+^, CD19^+^ cells in the draining lymph nodes (dLNs) and spleens from CD19^cre^AIM2^f/f^ mice and AIM2^f/f^ mice. **b** Representative flow cytometry diagrams and statistical analysis of CD4^+^ naive T cells, CD4^+^ memory T cells, CD8^+^ naive T cells, CD8^+^ memory T cells, CD19^+^CD44^+^ B cells, and CD19^+^CD62L^+^ B cells. **c** Representative flow cytometry diagrams and statistical analysis of naive B cells, memory B cells, plasmablast cells, plasma cells, GC B cells. Horizontal bars represent the mean ± SEM. *p < 0.05, **p < 0.01

Without any immunization, a reduced frequency of CD19^+^ B cells and an increased frequency of CD3^+^ T cells were observed in the draining lymph nodes (dLNs) and spleens of the CD19^cre^AIM2^f/f^ mice compared with the AIM2^f/f^ mice (Fig. 2a). In the dLNs and spleens from the CD19^cre^AIM2^f/f^ mice, we found an increased frequency of CD4^+^ naive T cells but a reduced frequency of CD4^+^ memory T cells and an increased frequency of CD62L^+^ B cells (inactive B cells) (Fig. 2b). Furthermore, in the spleens of the CD19^cre^AIM2^f/f^ mice, we observed a higher frequency of naive B cells and a lower frequency of memory B cells. In the dLNs of the CD19^cre^AIM2^f/f^ mice, we found increased frequencies of plasmablast cells and GC B cells (Fig. 2c). These data indicated that without AIM2 in B cells, B cells and T cells might display more stable and “naive” phenotypes.

### Reduced KLH-response and plasma cell proportion in AIM2 CKO mice

**Fig. 3:**
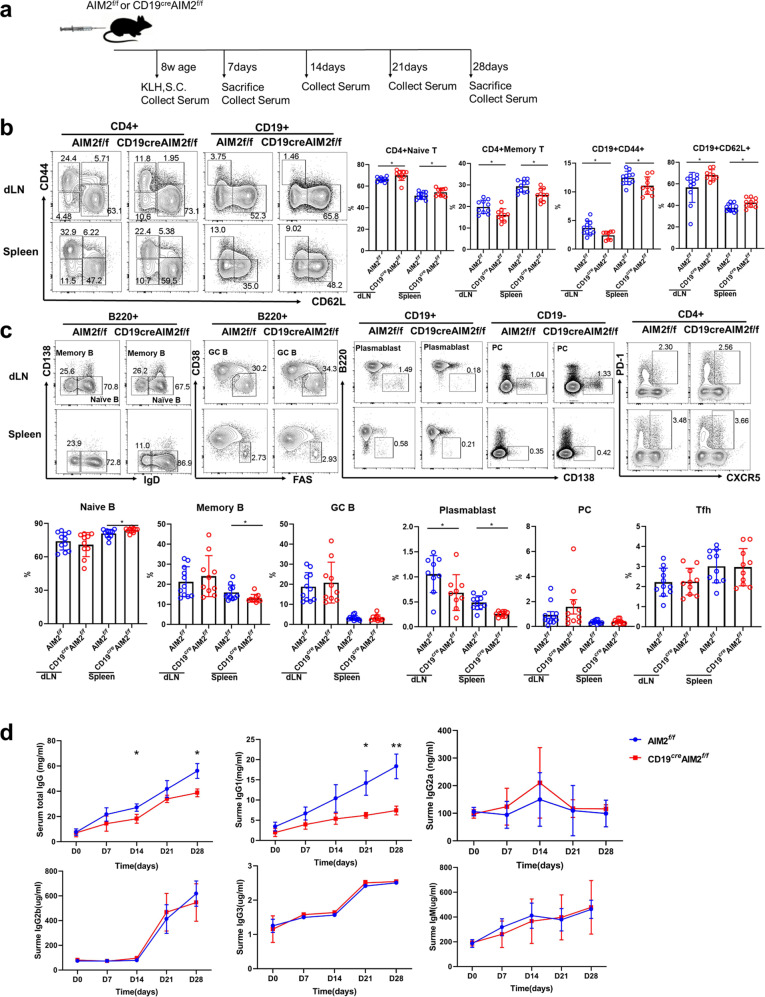
The KLH-induced response in CD19^cre^AIM2^f/f^ mice. **a** A schematic of the KLH-induced response experiments. On day 7 after KLH immunization, cells were collected from the dLNs and spleens of the CD19^cre^AIM2^f/f^ mice and AIM2^f/f^ mice. **b** Representative flow cytometry diagrams and statistical analysis of the percentages of CD4^+^ naïve T cells, CD4^+^ memory T cells, CD19^+^CD44^+^ B cells, and CD19^+^CD62L^+^ B cells in the dLNs and spleens of the CD19^cre^AIM2^f/f^ mice and AIM2^f/f^ mice. **c** Representative flow cytometry diagrams and statistical analysis of the percentages of naïve B cells, memory B cells, GC B cells, plasmablast cells, plasma cells, and Tfh cells in the dLNs and spleens of the CD19^cre^AIM2^f/f^ mice and AIM2^f/f^ mice. **d** The serum levels of total IgG, IgG1, IgG2a, IgG2b, IgG3, and IgM from the KLH-immunized mice on days 0, 7, 14, 21, and 28. Horizontal bars represent the mean ± SEM. *p < 0.05, **p < 0.01

To investigate the effect of AIM2 on the antigen-specific B-cell response, we conducted KLH immunization in the CD19^cre^AIM2^f/f^ mice and control mice (Fig. 3a). Immune cells, which were observed by flow cytometry, were collected on day 7 and day 28 after immunization. Serum samples were collected on days 7, 14, 21, and 28 after immunization. On day 7, consistent with the phenotype observations (described in Fig. 3), compared with the AIM2^f/f^ mice, the CD19^cre^AIM2^f/f^ mice showed higher frequencies of CD4^+^ naive T cells and inactivate B cells (CD19^+^CD62L^+^) but lower frequencies of CD4^+^ memory T cells and activated B cells (CD19^+^CD44^+^) (Fig. 3b). In addition, increased numbers of naive B cells but reduced numbers of memory B cells were found in the spleens of the CD19^cre^AIM2^f/f^ mice (Fig. 3c). Fewer plasmablast cells were found in the dLNs and spleens of the CD19^cre^AIM2^f/f^ mice, but no significant difference was found in the number of plasma cells (Fig. 3c). The numbers of cell types were shown in Supplementary Fig. 4 and the trend was similar with percentages.

T and B cells collected on day 28 produced similar results. As shown in Supplementary Fig. 5b, reduced frequencies of plasmablast cells, plasma cells, and T follicular helper (Tfh) cells were observed in the spleens of CD19^cre^AIM2^f/f^ mice. The decreased numbers of plasmablast cells, plasma cells, and Tfh cells were consistent with their frequencies (Supplementary Fig. 5c). More importantly, lower serum levels of total IgG and IgG1 were found in the CD19^cre^AIM2^f/f^ mice (Fig. 3d), indicating mild antibody production and antigen-specific responses in the AIM2 CKO mice.

### Attenuated lupus symptoms in AIM2 CKO mice

**Fig. 4:**
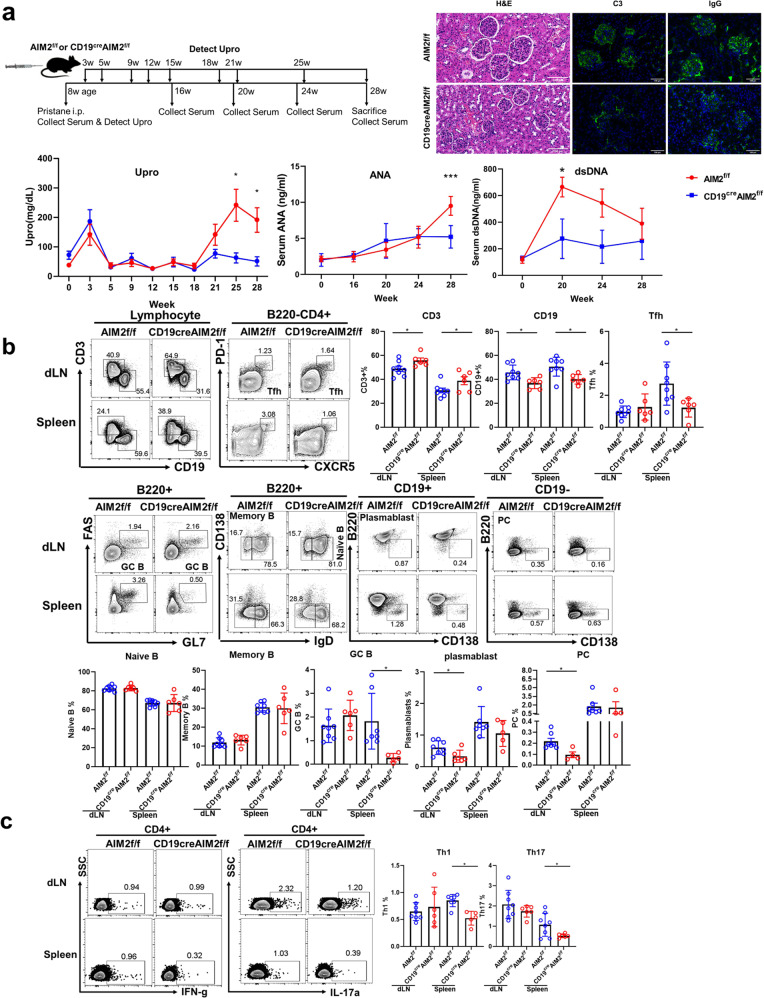
Pristane-induced lupus mouse in CD19^cre^AIM2^f/f^ mice. **a** A schematic of the induction of lupus in mice by pristane. H&E staining, C3 and IgG deposition in the kidney, proteinuria, and serum levels of ANAs and anti-dsDNA antibodies were shown. **b** Representative flow cytometry diagrams and statistical analysis of the frequencies of CD3 cells, CD19 cells, Tfh cells, naive B cells, memory B cells, GC B cells, plasmablast, and plasma cells in the dLNs and spleens of the CD19^cre^AIM2^f/f^ mice and AIM2^f/f^ mice. **c** Representative flow cytometry diagrams and statistical analysis of the frequencies of Th1 and Th17 cells in the dLNs and spleens of the CD19^cre^AIM2^f/f^ mice and AIM2^f/f^ mice. Horizontal bars represent the mean ± SEM. *p < 0.05, ***p < 0.005

As we observed greater numbers of AIM2^+^ B cells in SLE patients, we further investigated the effect of AIM2 in lupus disease progression. As shown in Fig. 4a, reduced proteinuria and serum levels of antinuclear antibodies (ANAs) and anti-dsDNA antibodies were found in the CD19^cre^AIM2^f/f^ pristane-induced lupus mice. Milder cell infiltration and less C3 and IgG deposition were observed in the kidneys of the CD19^cre^AIM2^f/f^ mice than in the kidneys of the AIM2^f/f^ mice (Fig. 4a). Fewer GC B cells and Tfh cells were found in the spleens of the AIM2 CKO mice. Fewer plasmablast cells and plasma cells were found in the dLNs of CD19^cre^AIM2^f/f^ mice (Fig. 4b). In addition, reduced frequencies of Th1 and Th17 cells were found in the spleens of AIM2 CKO mice (Fig. 4c), indicating that the loss of AIM2 in B cells can further regulate Tfh and effector T cells, leading to impaired inflammatory responses.

### AIM2 in B cells directly binds to Blimp-1 and regulates Blimp-1 and Bcl-6 expression

**Fig. 5:**
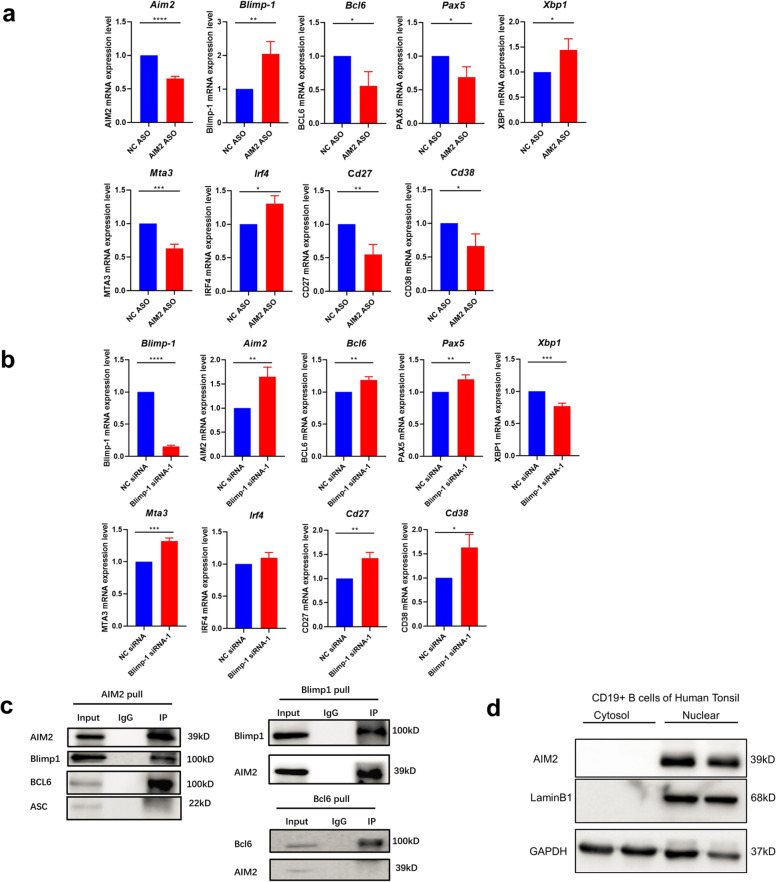
AIM2 regulates Blimp-1 and Bcl-6 expression and directly binds to Blimp-1 and Bcl-6. **a** AIM2 expression was knocked down in B cells by AIM2 ASO, and the mRNA expression of *Bcl6, Pax5, Xpb1, Mta3, Cd27*, *Cd38*, *Blimp-1*, and *Irf4* was detected by qRT-PCR. **b** Blimp-1 expression was knocked down in B cells by Blimp-1 siRNA, and the mRNA expression of *Bcl6, Pax5, Xpb1, Mta3, Cd27*, *Cd38*, *Aim2*, and *Irf4* was detected by qRT-PCR. **c** The Co-IP of AIM2 with Blimp-1, Bcl-6, and ASC. **d** The nuclear expression of AIM2 in human tonsil B cells. Horizontal bars represent the mean ± SEM. *p < 0.05, **p < 0.01, ***p < 0.005, ****p < 0.0001

Blimp-1 and Bcl-6 are transcription factors that are well documented to be key regulators of B-cell differentiation. Bcl-6 has been found to regulate GC B-cell differentiation, whereas Blimp-1 has been shown to cause plasma cell generation. As we observed that the AIM2 CKO mice showed a reduction in plasma cells, we further explored the AIM2-mediated regulatory pathway in B cells. AIM2 was expressed in the nucleus of human tonsil CD19^+^ B cells (Fig. 5d), as well as in the nucleus of peripheral naive B, memory B, and plasma cells (Supplementary Fig. 6). As shown in Fig. 5a, the silencing of AIM2 in human CD19^+^ B cells resulted in decreased mRNA expression of *Bcl6, Pax5, Mta3, Cd27*, and *Cd38* but increased expression of *Blimp-1*, *Xbp1,* and *Irf4*, suggesting that AIM2 might regulate B-cell differentiation via Bcl-6 and Blimp-1 regulation.

In addition, Blimp-1 was also silenced in B cells, and increased levels of *Aim2*, *Bcl6, Pax5, Mta3, Cd27*, and *Cd38* were found in Blimp-1 knockdown B cells (Fig. 5b). To explore the protein interactions, Co-IP was also conducted. AIM2 was found to directly interact with Blimp-1 and Bcl-6, while Blimp-1 exhibited a high affinity for AIM2, but Bcl-6 failed to pull down AIM2 (Fig. 5c). These findings indicated that AIM2 might positively regulate Bcl-6 expression and negatively regulate Blimp-1 expression via direct interaction. In addition, Blimp-1 can also negatively regulate AIM2 expression by direct interaction.

### IL-10 increases AIM2 expression via DNA demethylation

**Fig. 6:**
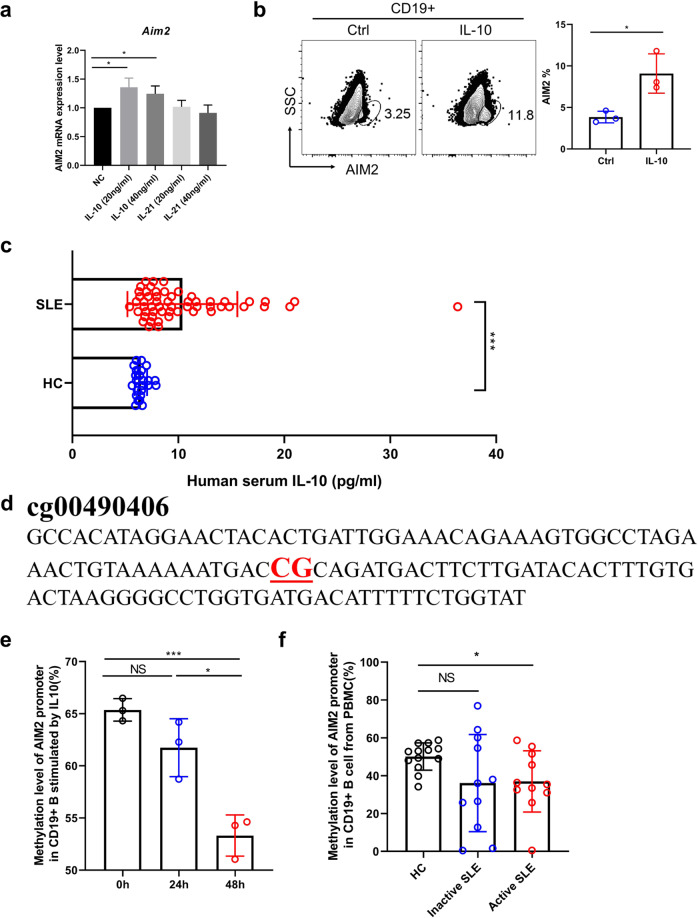
IL-10 upregulates AIM2 expression via DNA demethylation. **a** B cells were cultured with different concentrations of IL-10 and IL-21, and the mRNA expression of AIM2 was measured by qRT-PCR. **b** B cells were cultured with IL-10, and the expression of AIM2 was measured by flow cytometry. **c** The serum level of IL-10 from HC subjects (*n* = 24) and SLE patients (*n* = 56) was detected by ELISA. **d** The DNA sequence for the DNA methylation-sensitive region in the AIM2 promoter. **e** The DNA methylation level in the AIM2 promoter after IL-10 stimulation for 24 h and 48 h. **f** The DNA methylation level in the AIM2 promoter in B cells of SLE patients (*n* = 22) and HC subjects (*n* = 13). Horizontal bars represent the mean ± SEM. *p < 0.05, ***p < 0.005

To further identify the key factor involved in AIM2 expression in B cells, the potential role of the cytokines IL-10 and IL-21, which are key cytokines for B-cell differentiation, in AIM2 regulation was examined. As shown in Fig. 6a, IL-10 displayed a higher capacity to increase AIM2 expression. Consistent with other findings, a higher serum level of IL-10 was found in SLE patients (Fig. 6c). AIM2 has been reported to be regulated by DNA methylation modification. The DNA methylation-sensitive sequence was identified by our previous study and was shown in Fig. 6d. Unsurprisingly, IL-10 reduced the DNA methylation level of AIM2 (Fig. 6e), lupus B cells showed a lower level of DNA methylation of AIM2, and decreased DNA methylation was found in active SLE B cells (Fig. 6f). The stimulation of IL-10 increased the protein expression of AIM2 (Fig. 6b).

In order to investigate the effect of IL-10 on histone modification, we conducted ChIP-PCR analysis of H3AC, H3K4me3, H3K9me3, H3K27me3 on AIM2 promoter region, with or without IL-10 treatment and in NC vs active and inactive SLE patients (Supplementary Fig. 7). The enrichment of H3K4me3 was significantly increased on the promoter region position 1 in inactive and active patients, as well as on position 3 in inactive SLE patients (Supplementary Fig. 7c). IL-10 treatment can slightly increase the H3K27me3 levels on positions 1, 2, 3, 4, but no significant difference was detected (Supplementary Fig. 7b). Therefore, our results indicated that histone modification did not contribute to the AIM2 expression in this scenario.

## Discussion

In cancer studies, AIM2 is capable of inducing antigen-specific antibody response and has been proposed as an adjuvant to enhance therapeutic efficacy via CD8^+^ T cells.^13–15^ This antigen-specific adaptive immune response is reduced in AIM2 KO mice after DNA vaccination, which is in IL-1beta and IL-18 independent manner, suggesting an inflammasome-independent function of AIM2 in adaptive immune cells. In healthy people, preferential expression of AIM2 is described in memory CD27^+^ B cells.^16^ AIM2 is also described in gastric B cells and is responsible for the CXCL16 expression in gastric B cells.^17^ In addition, a study reported that AIM2 was directly repressed by FOXP1 in human B cells.^18^ This evidence indicates that AIM2 is highly expressed in B cells but the function and regulatory mechanisms are largely unknown.

AIM2 is described as an IFN-inducing gene. Interestingly, SLE is reported as a type I IFN associated disease. Therefore, copied studies have reported the positive association between AIM2-inflammasome and lupus pathogenesis.^19,20^ Increased expression level of AIM2 was observed in lupus livers, spleens, and peripheral blood mononuclear cells (PBMCs), and enhanced activation of AIM2-inflammasome was found in lupus unstimulated macrophages.^20^ More interestingly, a reduction of DNA methylation of AIM2 was reported in lupus patients compared with their healthy siblings.^21^ All the evidence mentioned above suggests that AIM2 is abnormally expressed in lupus immune cells and regulated by DNA methylation. However, the mechanisms are unclear.

In this study, we first described the high expression of AIM2 in human B cells and B-cell subtypes, especially in differentiated B cells, but no ASC was found to bind to AIM2 (Fig. 5c). Except for naive B cells, memory B cells and plasma cells from lupus patients showed higher expression of AIM2 compared with HCs, suggesting that AIM2 might contribute to the pathogenesis of SLE. SLE is reported to be mediated by auto-antibodies and B cells. As the key transcription factors, Blimp-1 and Bcl-6 orchestrate the differentiation of GC B cells and plasma cells, and these two transcription factors suppress each other by histone modifications, which makes them an axis. In our findings, AIM2 deficiency led to the downregulation of Bcl-6 and PAX5 expression, but upregulated the expression of Blimp-1, XBP1, and IRF4, suggesting that AIM2 regulates B-cell differentiation via Blimp-1-Bcl-6 axis.

PAX5 is highly expressed by follicular B cells, activated B cells, GC B cells, and memory B cells. In the differentiation of B cells, follicular B cells are long-lived and quiescent B cells and they will differentiate into short-lived and cycling activated B cells, which can further differentiate into either short-lived plasmablast cells (Blimp-1^mid^) or short-lived and rapidly cycling GC B cells (Bcl-6^hi^). GC B cells will then either lose the expression of Bcl-6 and highly express Blimp-1, further differentiating into long-lived plasma cells (Blimp-1^hi^, IRF4^hi^, XBP1^hi^), or differentiate into long-lived memory B cells (PAX5^hi^).^22^ This means that although Blimp-1 and Bcl-6 suppress each other, GC B cells are the source of plasma cells, and activated B cells are the source of plasmablast and GC B cells, and the number of plasma cells still relies on the number of GC B cells and activated B cells. If we look at the whole immune system, a reduced number of activated B cells and GC B cells will lead to a reduced number of plasma cells and plasmablast cells. Indeed, in our KLH model and pristane-induced lupus model, the loss of AIM2 in B cells finally resulted in the reduced frequencies of GC B cells, Tfh cells, plasmablast cells, and plasma cells.

To explore the upregulator of AIM2 in lupus, IL-21 and IL-10 are considered. IL-21 and IL-10 are co-regulators for antibody production. In this study, we found that IL-10 was capable of inducing AIM2 expression in B cells. IL-10 is well known as an upregulator for B-cell growth and differentiation, proliferation, class-switching, and IgG production.^23^ IL-10 is reported to be highly expressed in lupus serum and positively correlated with disease activity and IgG production.^24^ In addition, IL-10 is identified as a pathogenic factor in many lupus mouse models.^24,25^ This evidence indicates that in SLE patients, IL-10 serves as a pathogenic role rather than an anti-inflammatory factor. Consistently, we also observed increased serum levels of IL-10, which can enhance AIM2 expression via DNA demethylation. This finding might be an explanation for the increased expression of AIM2 in B cells from lupus peripheral blood.

AIM2 is not a CpG island gene, however, AIM2 has been found to be regulated by DNA methylation.^26^ In addition, we did the DNA methylation map (450K) in T cells, and three CpG sites have been identified in the AIM2 promoter region. We designed the primers to use pyrosequencing to observe the methylation of these CpG sites. However, only one CpG site showed a significant difference, in the comparison of SLE patients with HCs (Fig. 6). Although only a single CpG site has been identified in our study, a single CpG site has been also reported to regulate gene expression in genes. For example, the DNA methylation on a single CpG site from *Il6* promoter was reported to be related with Il6 mRNA expression levels,^27^ and single CpG hypermethylation was also reported to be responsible for the decreased tumor suppressor gene expression in cancers.^28^ Therefore, we believed that the DNA methylation level on this single CpG site might contribute to the AIM2 overexpression in lupus B cells.

Together, in this study, we demonstrated a novel function of AIM2 in B cells, described an IL-10-DNA demethylation-AIM2-Blimp-1/Bcl-6 pathway, and revealed the pathogenic role of AIM2 in antibody production, providing a potential therapeutic target for SLE treatment, and shedding light on the pathogenesis and therapy of humoral mediated autoimmune diseases.

## Materials and methods

### Patients

22 human tonsil tissues were ground and filtered to obtain a single-cell suspension to be used to analyze the expression level of AIM2, Blimp-1, and Bcl-6 in each subset of B cells. Isolating peripheral blood mononuclear cells (PBMCs) from 56 SLE patients and 70 healthy controls (HCs) to analyze the AIM2 expression level in B-cell subsets. Isolating CD19^+^ B cells from PBMCs of SLE patients and HCs to get total genome DNA, which are used to detect the methylation level of AIM2 promoter by pyrosequencing. Using multi-immunohistochemistry (multi-IHC) staining technique to analyze AIM2 expression level in CD19^+^ B cells from SLE and DLE skin lesions and HC tonsil tissues. Serums from 56 SLE patients and 24 HCs were used to detect the level of IL-10 by ELISA.

Naive B cells from the peripheral blood of HCs were isolated by human naive B-cell magnetic beads. 2 × 10^6^ cells per well were seeded in a 24-well plate stimulated with IL-10 (20 ng/ml). After 48 h of stimulation, samples were collected to analyze the expression levels of AIM2 in B cells by flow cytometry. Additionally, CD19^+^ B cells from PBMCs of HCs stimulated with IL-10 (20 ng/ml) after 24 and 48 h were used to analyze the methylation level of AIM2 promoter by pyrosequencing.

### Animal models

#### KLH immunization

CD19^cre^AIM2^f/f^ and AIM2^f/f^ mice (8 weeks old) were subcutaneously immunized with equal amounts (400 μl per mouse) of KLH (0.5 mg/ml, Sigma), which was emulsified in CFA (0.5 mg/ml, Sigma), on the tail. After immunization, the mice were sacrificed, and the lymph nodes, spleen tissues, and serum samples were collected. The lymph nodes and spleen tissues were analyzed by flow cytometry. The serum antigen-specific total IgG, IgG1, IgG2a, IgG2b, IgG3, and IgM antibody levels were measured by ELISA.

#### Pristane-induced lupus-like mouse model

Female CD19^cre^AIM2^f/f^ and AIM2^f/f^ mice aged 8-10 weeks were intraperitoneally injected with 500 μl pristane (Sigma) per mouse. Urine samples were collected from the pristane-treated mice, and proteinuria was assessed by a colorimetric assay strip (URIT). The serum samples of pristane-treated mice were collected at the beginning and the end of the observation period. The serum anti-dsDNA IgG and ANA IgG levels were detected by ELISA.

Draining lymph nodes (dLNs) and spleens from two models were used to execute immune cell analysis by flow cytometry. Using multi-IHC staining technique to analyze the C3 and IgG deposition in kidneys of pristane model.

### Co-IP and western blotting

Nuclear and cytosolic proteins were extracted in CD19^+^ B cells from human tonsils and spleens of the animal model by using a kit (Invent SC-003), and used to analyze the location of AIM2 by western blotting. Additionally, extracting the total proteins of CD19^+^ B cells from human tonsils to explore the interaction of AIM2 with Blimp-1, Bcl-6, and ASC by Co-IP.

### Silencing AIM2 or Blimp-1 in vitro

First, we sorted CD19^+^ B cells from healthy subjects with magnetic beads (Miltenyi Biotec, 130-050-301). Second, 2 × 10^6^ cells were resuspended in 100 µl electroporation liquid (Lonza, V4XP-3024) and then mixed with 2.5 µL AIM2 ASO (Ruibo, 20 µM) or 2.5 µl Prdm1 siRNA (Ruibo, 20 µM). All of the cells were transferred into electroporation cups to be transfected following the human B-cell protocol in an electrical transfection instrument (Lonza, 4D-Nuclefector^TM^). Finally, all the transfected cells were transferred into 1 ml of 1640 complete culture medium (90% 1640 with 10% FBS) containing IL-10 as a stimulator (the final concentration was 20 ng/ml) and cultured in an incubator (37 °C, 5% CO_2_). After 48 h, total RNA from all the samples was collected by TRIzol for subsequent qRT-PCR analysis.

All methods are described in detail in the Supplementary Material.

### Statistical analysis

We used SPSS 18.0 to perform the statistical analyses. All the data were presented as the mean ± SEM and were assessed for normal distribution and similar variance between groups. Statistical significance of groups was assessed using two-tailed unpaired Student’s *t*-tests for comparisons between two groups and one-way analysis of variance (ANOVA) with relevant post-hoc tests for multiple comparisons. We used the two-tailed Mann-Whitney *U* test for statistical analyses when the data were not normally distributed or displayed unequal variances between two groups. The correlation analysis of two indexes was performed using Pearson’s r test or Spearman’s *r* test (for abnormally distributed data). No statistical method was used to predetermine the sample size. All the animals were randomly divided into treatment groups.

### Study approval

All human sample studies were approved by the ethics committee of the Second Xiangya Hospital of Central South University. We obtained written informed consent from all the subjects. All the animal care protocols and experiments were reviewed and approved by the Animal Care and Use Committee of the Laboratory Animal Research Center at the Second Xiangya Hospital of Central South University.

## Supplementary information

Supplementary Materials

## Data Availability

All data supporting this paper are present within the paper and/or the Supplementary Materials. The original data sets are also available from the corresponding author upon request.

## References

[1] DeYoung KL (1997). Cloning a novel member of the human interferon-inducible gene family associated with control of tumorigenicity in a model of human melanoma. Oncogene.

[2] Cridland JA (2012). The mammalian PYHIN gene family: phylogeny, evolution and expression. BMC Evol. Biol..

[3] Roberts TL (2009). HIN-200 proteins regulate caspase activation in response to foreign cytoplasmic DNA. Science.

[4] Hornung V (2009). AIM2 recognizes cytosolic dsDNA and forms a caspase-1-activating inflammasome with ASC. Nature.

[5] Fernandes-Alnemri T (2009). AIM2 activates the inflammasome and cell death in response to cytoplasmic DNA. Nature.

[6] Burckstummer T (2009). An orthogonal proteomic-genomic screen identifies AIM2 as a cytoplasmic DNA sensor for the inflammasome. Nat. Immunol..

[7] Wilson JE (2015). Inflammasome-independent role of AIM2 in suppressing colon tumorigenesis via DNA-PK and Akt. Nat. Med..

[8] Tsokos GC (2011). Systemic lupus erythematosus. N. Engl. J. Med..

[9] Wu H (2018). Epigenetic regulation in B-cell maturation and its dysregulation in autoimmunity. Cell Mol. Immunol..

[10] Dogan I (2009). Multiple layers of B cell memory with different effector functions. Nat. Immunol..

[11] Ochiai K (2008). Regulation of the plasma cell transcription factor Blimp-1 gene by Bach2 and Bcl6. Int Immunol..

[12] Shapiro-Shelef M, Calame K (2005). Regulation of plasma-cell development. Nat. Rev. Immunol..

[13] Chai D (2015). AIM2 co-immunization favors specific multifunctional CD8(+) T cell induction and ameliorates coxsackievirus B3-induced chronic myocarditis. Antivir. Res..

[14] Yin L (2017). AIM2 co-immunization with VP1 is associated with increased memory CD8 T cells and mounts long lasting protection against coxsackievirus B3 challenge. Front. Cell. Infect. Microbiol..

[15] Chai D (2019). Combining DNA vaccine and AIM2 in H1 nanoparticles exert anti-renal carcinoma effects via enhancing tumor-specific multi-functional CD8(+) T-cell responses. Mol. Cancer Ther..

[16] Svensson A (2017). Maturation-dependent expression of AIM2 in human B-cells. PLoS ONE.

[17] El-Zaatari, M. et al. Aim2-mediated/IFN-beta-independent regulation of gastric metaplastic lesions via CD8+ T cells. *JCI Insight* **5**, e94035 (2020). 10.1172/jci.insight.94035 PMC7141403 32053518

[18] van Keimpema M (2014). FOXP1 directly represses transcription of proapoptotic genes and cooperates with NF-kappaB to promote survival of human B cells. Blood.

[19] Ding L (2015). The regional function of cGAS/STING signal in multiple organs: one of culprit behind systemic lupus erythematosus?. Med. Hypotheses.

[20] Yang CA, Huang ST, Chiang BL (2015). Sex-dependent differential activation of NLRP3 and AIM2 inflammasomes in SLE macrophages. Rheumatology.

[21] Javierre BM (2010). Changes in the pattern of DNA methylation associate with twin discordance in systemic lupus erythematosus. Genome Res..

[22] Nutt SL, Hodgkin PD, Tarlinton DM, Corcoran LM (2015). The generation of antibody-secreting plasma cells. Nat. Rev. Immunol..

[23] Ma CS (2005). Impaired humoral immunity in X-linked lymphoproliferative disease is associated with defective IL-10 production by CD4+ T cells. J. Clin. Investig..

[24] Cairns AP, Crockard AD, Bell AL (2003). Interleukin-10 receptor expression in systemic lupus erythematosus and rheumatoid arthritis. Clin. Exp. Rheumatol..

[25] Ishida H (1994). Continuous administration of anti-interleukin 10 antibodies delays onset of autoimmunity in NZB/W F1 mice. J. Exp. Med..

[26] Yamazaki J (2012). Effects of TET2 mutations on DNA methylation in chronic myelomonocytic leukemia. Epigenetics.

[27] Nile CJ (2008). Methylation status of a single CpG site in the IL6 promoter is related to IL6 messenger RNA levels and rheumatoid arthritis. Arthritis Rheum..

[28] Böck J (2018). Single CpG hypermethylation, allele methylation errors, and decreased expression of multiple tumor suppressor genes in normal body cells of mutation-negative early-onset and high-risk breast cancer patients. Int J. Cancer.

