# Supplementary material for: AIM2 deficiency in B cells ameliorates systemic lupus erythematosus by regulating Blimp-1–Bcl-6 axis-mediated B-cell differentiation
Source: Signal Transduct Target Ther. 2021 Sep 14;6:341. doi: 10.1038/s41392-021-00725-x (PMC8440614; doi:10.1038/s41392-021-00725-x)
Supplement: Supplementary file 1 — Supplementary Materials [file 41392_2021_725_MOESM1_ESM.pdf]

Supplementary Materials for

AIM2 Deficiency in B Cells Ameliorates Systemic Lupus Erythematosus by Regulating Blimp-1-Bcl-6 Axis Mediated-B Cell Differentiation

Ming Yang1, Di Long1, Longyuan Hu1, Zhidan Zhao1, Qianwen Li1, Yunkai Guo3, Zhenghao He1, Ming Zhao1, Liwei Lu4, Fen Li5, Hai Long1, Haijing Wu*1, Qianjin Lu*1,2

Correspondence to: Qianjin Lu: qianlu5860@csu.edu.cn; Haijing Wu: chriswu1010@csu.edu.cn

Materials and Methods

Patients

22 human tonsil tissues were ground and filtered to obtain a single-cell suspension to be used to analyze the expression level of AIM2, Blimp-1 and Bcl-6 in each subset of B cells. Isolating peripheral blood mononuclear cells (PBMCs) from 56 SLE patients and 70 healthy controls (HCs) to analyze the AIM2 expression level in B cell subsets. Isolating $CD19^+$ B cells from PBMCs of SLE patients and HCs to get total genome DNA which are used to detect the methylation level of AIM2 promoter by pyrosequencing. Using multi-immunohistochemistry (multi-IHC) staining technique to analyze AIM2 expression level in $CD19^+$ B cells from SLE and DLE skin lesions and HC tonsil tissues. Serums from 56 SLE patients and 24 HCs were used to detect the level of IL-10 by ELISA.

Naïve B cells from the peripheral blood of HCs were isolated by human naïve B cell magnetic beads. $2x10^6$ cells per well were seeded in a 24-well plate stimulated with IL-10 (20ng/ml). After 48 hours of stimulation, samples were collected to analyze the expression levels of AIM2 in B cells by flow cytometry. Additionally, $CD19^+$ B cells from PBMCs of HCs stimulated with IL-10(20ng/ml) after 24h and 48h were used to analyze the methylation level of AIM2 promoter by pyrosequencing.

Animal models

KLH-immunization: $CD19^{cre}AIM2^{f/f}$ and $AIM2^{f/f}$ mice (8 weeks old) were subcutaneously immunized with equal amounts (400 µl per mouse) of KLH (0.5 mg/ml, Sigma), which was emulsified in CFA (0.5 mg/ml, Sigma), on the tail. After immunization, the mice were sacrificed, and the lymph nodes, spleen tissues, and serum samples were collected. The lymph nodes and spleen tissues were analyzed by flow cytometry. The serum antigen-specific total IgG, IgG1, IgG2a, IgG2b, IgG3, and IgM antibody levels were measured by ELISA.

Pristane-induced lupus-like mouse model: Female CD19^{cre}AIM2$^{f/f}$ and AIM2$^{f/f}$ mice aged 8-10 weeks were intraperitoneally injected with 500µl pristane (Sigma) per mouse. Urine samples were collected from the pristane-treated mice, and proteinuria was assessed by a colorimetric assay strip (URIT). The serum samples of pristane-treated mice were collected at the beginning and the end of the observation period. The serum anti-dsDNA IgG and ANA IgG levels were detected by ELISA.
Draining lymph nodes (dLNs) and spleens from two models were used to execute immune cell analysis by flow cytometry. Using multi-IHC staining technique to analyze the C3 and IgG deposition in kidneys of pristane model.

Co-IP and western blotting

Nuclear and cytosolic proteins were extracted in CD19$^+$ B cells from human tonsils and spleens of animal model by using a kit (Invent SC-003), and used to analyze the location of AIM2 by western blotting. Additionally, extracting the total proteins of CD19$^+$ B cells from human tonsils to explore the interaction of AIM2 with Blimp-1, Bcl-6 and ASC by Co-IP.

Silencing AIM2 or Blimp-1 in vitro

First, we sorted CD19$^+$ B cells from healthy subjects with magnetic beads (Miltenyi Biotec, 130-050-301). Second, 2x10^6 cells were resuspended in 100 µL electroporation liquid (Lonza, V4XP-3024) and then mixed with 2.5 µL AIM2 ASO (Ruibo, 20 µM) or 2.5 µL Prdm1 siRNA (Ruibo, 20 µM). All of the cells were transferred into electroporation cups to be transfected following the human B cell protocol in an electrical transfection instrument (Lonza, 4D-Nuclefector™). Finally, all the transfected cells were transferred into 1 mL of 1640 complete culture medium (90% 1640 with 10% FBS) containing IL-10 as a stimulator (the final concentration was 20 ng/ml) and cultured in an incubator (37°C, 5% CO_2). After 48 hours, total RNA from all the samples was collected by TRIzol for subsequent qRT-PCR analysis.

All methods are described in detail in the supplementary material.

Statistical analysis

We used SPSS 18.0 to perform the statistical analyses. All the data were presented as the mean ± SEM and were assessed for normal distribution and similar variance between groups. Statistical significance of groups was assessed using two-tailed unpaired Student's t-tests for comparisons between two groups and one-way analysis of variance (ANOVA) with relevant post-hoc tests for multiple comparisons. We used the 2-tailed Mann-Whitney U test for statistical analyses when the data were not normally distributed or displayed unequal variances between two groups. The correlation analysis of two indexes was performed using Pearson's r test or Spearman's r test (for abnormally distributed data). No statistical method was used to predetermine the sample size. All the animals were randomly divided into the treatment groups.

Study approval

All human sample studies were approved by the ethics committee of the Second Xiangya Hospital of Central South University. We obtained written informed consent from all the subjects. All the animal care protocols and experiments were reviewed and approved by the Animal Care and Use Committee of the Laboratory Animal Research Center at the Second Xiangya Hospital of Central South University.

Supplementary Methods

Subjects

Tonsil flow cytometry and immunohistochemistry (IHC) samples: 22 clinical samples of human tonsil tissues were collected from Department of Otolaryngology head and neck surgery of the Second Xiangya hospital of Central South University. The detailed information of patients is listed in Supplemental Table 1.

PBMC flow cytometry samples: 56 SLE patients who fulfilled at least 4 of the SLE classification criteria of the American College of Rheumatology were recruited from outpatient clinics of the Second Xiangya Hospital of Central South University. Lupus disease activity was assessed using the **SLE Disease Activity Index (SLEDAI)**. 70 sex-and age-matched healthy controls (HCs) were recruited from the Physical Examination Center at the Second Xiangya Hospital of Central South University. The detailed information of SLE patients and HCs is listed in Supplemental Table 2.

Pyrosequencing samples: 21 SLE patients were recruited from outpatient clinics of the Second Xiangya Hospital of Central South University. 13 sex-and age-matched HCs were recruited from the Physical Examination Center at the Second Xiangya Hospital of Central South University. The detailed information of SLE patients and HCs is listed in Supplemental Table 3.

Lupus skin lesion samples: 3 SLE and 3 DLE patients' skin lesion tissues were collected from the department of dermatopathology of the Second Xiangya Hospital of Central South University. The detailed information of SLE patients and HCs is listed in Supplemental Table 4.

Mice

AIM2$^{f/f}$ mice and AIM2$^{-/-}$ mice were purchased from the Shanghai Model Organisms Company. C57BL/6J mice were purchased from the Slack Company. CD19^{cre} mice were donated by Professor Wang Honglin of Shanghai Jiaotong University. AIM2$^{f/f}$ mice were bred with CD19^{cre} mice to generate control AIM2$^{f/f}$ and CD19^{cre}AIM2$^{f/f}$ (CKO) mice.

KLH immunization

CD19^{cre}AIM2$^{f/f}$ and AIM2$^{f/f}$ mice (8 weeks old) were subcutaneously immunized (400μl per mouse) with KLH (0.5 mg/ml, Sigma) which was emulsified in CFA (0.5 mg/ml, Sigma) on the tail equally. After immunization, mice were sacrificed, and lymph nodes, spleen tissues and serum samples were collected. Lymph nodes and spleen tissues were analyzed by flow cytometry. Serum antigen-specific total IgG (Alpha Diagnostic Intl, cat.6320), IgG1(Alpha Diagnostic Intl, cat.6330), IgG2a (Alpha Diagnostic Intl, cat.6340), IgG2b (Alpha Diagnostic Intl, cat.6350), IgG3(Alpha Diagnostic Intl, cat.6360), and IgM (4Abio, CME0060-096) antibody levels were measured by ELISA.

Pristane-induced lupus-like mouse model

Female $CD19^{cre}AIM2^{f/f}$ and $AIM2^{f/f}$ mice aged 8-10 weeks were injected intraperitoneally with 500μl pristane (Sigma) per mouse. Pristane models' urine samples were collected, and proteinuria was assessed by a colorimetric assay strip (URIT). Proteinuria assessed by the following scale: Absent=0mg·dl^{-1}; ±=15mg·dl^{-1}; +=30mg·dl^{-1}; ++=100mg·dl^{-1}; +++=300mg·dl^{-1}; and ++++≥500mg·dl^{-1}. Serum samples of pristane models were collected at the start and the end of the observation period. Serum anti-dsDNA IgG (CUSABIO, cat. CSB-E11194m) and ANA IgG (CUSABIO, cat. CSB-E12912m) were detected by ELISA.

Human serum IL-10 ELISA analysis

Collecting the serum samples of 56 SLE patients who were recruited from outpatient clinics of the Second Xiangya Hospital of Central South University and 24 sex-and age-matched healthy controls (HC) who were recruited from the Physical Examination Center at the Second Xiangya Hospital of Central South University. Serum IL-10 from SLE patients and HCs were analyzed by ELISA according to the protocol (4Abio, cat. CHE0013-096). The detailed information of SLE patients and HCs is listed in Supplemental Table5.

Human naïve B cells were stimulated with IL-10 in vitro

Naïve B cells from peripheral blood of healthy people were isolated by human naïve B cell magnetic beads (Miltenyi Biotec, cat. 130-091-150). $2x10^6$ cells per well of 24-well plate were stimulated with IL-10 (Sino Biological, cat. 10947-HNAE, 20ng/ml) respectively. After 48 hours of stimulation, samples were collected to analyze the expression levels of AIM2 in each subgroup of B cells (including naïve B cells, memory B cells and plasma cells) by flow cytometry. The detailed information of healthy donors is listed in Supplemental Table6.

Flow cytometry analysis

Human PBMCs and Tonsil tissues: Peripheral blood mononuclear cells (PBMCs) were separated by Ficoll-Paque Plus (GE healthcare, cat. 17-1440-03) to get a single-cell suspension. Human tonsil tissues were ground and filtered to obtain a single-cell suspension. About $2×10^6$ cells were suspended in PBS and incubated with human Fc-R block (BD Pharmingen, 564765) at room temperature for 10 minutes, and then incubated with antibodies against surface markers at 4°C for 45 minutes in the dark. For intracellular staining, cells were fixed and permeabilized with a Human Foxp3 Buffer Set (BD Bioscience, 560098) and then stained with fluorescent antibodies for an additional 60 minutes at 4°C in the dark. All events were collected and analyzed by FlowJo software. The following antibodies were used: Zombie Aqua™ Fixable Viability Kit (Biolegend, 423102), APC anti-human CD19 (BD Pharmingen, clone HIB19, cat.555415), APC/Cy7 anti-human CD20 (Biolegend, clone 2H7, cat.302314), BB515 anti-human CD27(BD Horizon, clone M-T271, cat.564642), PerCP-Cy™5.5 anti-human CD38 (BD Pharmingen, clone HIT2, cat.551400), BV421 anti-human IgM(BD Horizon, clone G20-127, cat.562618), PE-Cy™7 anti-human IgD (BD Pharmingen, clone IA6-2, cat.561314), PE anti-human AIM2 (Biolegend, clone 3B10, cat.652804), PE anti-human Blimp-1 (BD Pharmingen, clone 6D3, cat.564702). All samples were detected by Flow Cytometry DxP Athena™ (America, Cytek).

Lymph nodes and Spleens from CD19^{cre}AIM2$^{f/f}$ and AIM2$^{f/f}$ mice (non-and KLH-immunization)

dLNs and spleen tissues were ground and filtered to obtain a single-cell suspension. Approximately 1×10^6 cells were suspended in PBS and incubated with mouse Fc-R block (BD Pharmingen, 553141) at room temperature for 10 minutes, and then incubated with antibodies against surface markers at 4°C for 45 minutes in the dark. For intracellular staining, cells were fixed and permeabilized with a Transcription Factor Buffer Set (BD Pharmingen, 562574) and then stained with fluorescent antibodies for an additional 60 minutes at 4°C in the dark. Additionally, before the flow cytometry analysis of cytokines of lymphocytes from dLNs and spleens, 2×10^6 cells/well were cultured and stimulated with the Leukocyte Activation Cocktail (BD GolgiPlug™ antibody, BD 550583) in 6-well plates in a cell incubator for 4 hours (37°C, 5% CO_2). All events were collected and analyzed by FlowJo software. The following antibodies were used: Zombie Aqua™ Fixable Viability Kit (Biolegend, 423102), FITC Rat anti-mouse B220 (BD Pharmingen, Clone RA3-6B2, cat.553088), PerCP/Cy5.5 Rat anti-mouse IgD (Biolegend, Clone 11-26c.2a, cat. 405710), BV421 Rat anti-Mouse CD138 (BD Horizon, Clone 281-2, cat. 562610), PE Hamster anti-mouse CD95 (BD Bioscience, clone Jo2, cat. 554258), PE/Cy7 anti-mouse CD38 (Biolegend, clone 90, cat.102718), BV711 anti-mouse CD19 (Biolegend, clone 6D5, cat. 115555), FITC Hamster anti-mouse CD3e (BD Pharmingen, clone 145-2c11, Cat. 553062), APC/Cy7 Rat anti-mouse CD4 (BD Pharmingen, clone GK1.5, cat. 552051), PerCP/Cy5.5 Rat anti-mouse CD8a (BD Pharmingen, clone 53-6.7, cat. 551162), APC Rat anti-mouse CD19 (BD Horizon, clone 1D3, cat.550992), PE Rat anti-mouse CD62L (BD Pharmingen, clone MEL-14, cat.561918), PE/Cy7 Rat anti-mouse CD44 (BD Pharmingen, clone IM7, cat. 560569), APC anti-mouse CD4 (Biolegend, clone GK1.5, cat. 100412), PE Hamster anti-mouse CD279 (BD Pharmingen, clone J43, cat. 551892), purified Rat anti-mouse CXCR5 (BD Pharmingen, cat. 551961), FITC goat anti-rat IgG2a secondary antibody (Invitrogen, cat. 800-874-3723), AF647 Rat anti-mouse T-and B-cell Activation Antigen (BD Pharmingen, clone GL7, cat. 561529), BV605 anti-mouse/human B220 (Biolegend, clone RA5-6B2, cat. 103244), BV421 anti-mouse CD4 (Biolegend, clone GK1.5, cat.100438), Biotin Rat anti-mouse CXCR5 (BD Pharmingen, cat.551960), PE streptavidin (BD Pharmingen, cat.554061), APC Hamster anti-mouse CD279 (BD Pharmingen, clone J43, cat.562671), AF488 anti-mouse IgD (Biolegend, clone 11-26c-2a, cat.405718), PE/Cy7 Rat anti-mouse CD19 (BD Pharmingen, clone 1D3, cat.552854), BB515 Rat anti-mouse CD138 (BD Horizon, clone 281-2, cat.564511), PerCP- Cy5.5 anti-mouse CD4 (Biolegend, clone GK1.5, cat 100434), APC Rat anti-mouse B220 (BD Pharmingen, clone RA3-6B2, cat.553092). All samples were detected by Flow Cytometry DxP Athena™ (America, Cytek).

Absolute cells count of B cells by flow cytometry

All gating cells of absolute counts were got by flowing calculation formula: Cells (counts)= Gated-1 (percent of lymphocytes) *Gated-2 (percent of B220$^+$/CD19$^+$ B cells/CD4$^+$ T cells) *Gated-3 (targeted cells) *10^6/volume(ml).

Nucleus-cytosol extraction and western blotting

Nuclear and cytosolic proteins were extracted from human tonsil $CD19^+$ B cells by using a kit (Invent SC-003). Also, nuclear and cytosolic proteins of $CD19^+$ B cells of spleen tissues from $CD19^{cre}AIM2^{f/f}$, $AIM2^{f/f}$, $AIM2^{-/-}$ (KO) and C57BL/6J mice were extracted by using a kit (Invent SC-003). Nuclear and cytosolic proteins or total proteins of $CD19^+$ B cells of human or mice were detected by western blotting. The following antibodies were used: AIM2 (Human, Abcam, ab204995, 1:1000), LaminB1 (CST, 13435S, 1:1000), GAPDH (Proteintech, 10494-1AP, 1:5000), AIM2(Mouse Specific, CST, 63660S, 1:1000), HRP-linked anti-rabbit IgG (CST, 7074S, 1:5000), HRP-linked Anti-mouse IgG (CST, 7076S, 1:5000).

Co-IP (AIM2 & Blimp1 & Bcl6 & ASC)

We collected and extracted protein of the treated fresh $CD19^+$ B cells from human tonsil tissues using RIPA lysis buffer (Beyotime, cat. P0013B) supplemented with PMSF (Beyotime, cat. ST506). Co-IP assays were performed with the Dynabeads Protein G IP Kit (Invitrogen, cat. 10007D) based on the manufacturer's protocols. The presence or absence of the target protein, the endpoint of the assay, was evaluated by western blotting. The following antibodies for IP were used: anti-AIM2 (CST, clone D5X7K, cat.12948S, 1:1000), anti-Blimp-1 (CST, clone C14A4, cat.9115S, 1:1000), anti-Bcl6 (CST, clone D56C10, cat.5650S, 1:1000) and anti-ASC (CST, clone E1E3I, cat.13833, 1:1000), HRP-linked anti-rabbit IgG (CST, cat. 7074S, 1:5000).

ASO RNA, siRNA and transfection

Firstly, we sorted $CD19^+$ B cells from healthy human by magnetic beads (Miltenyi Biotec, 130-050-301). Secondly, $2x10^6$ cells were resuspended into 100µL electroporation liquid (LONZA, V4XP-3024), and then mixed with 2.5µL AIM2 ASO (Ruibo, 20µM) or 2.5µL Prdm1 siRNA (Ruibo, 20µM). All of them were transferred into electroporation cups to be transfected by executing human B cells protocol in the electrical transfection instrument (LONZA, 4D-Nuclefector™). Finally, all transfected cells were transferred into 1mL1640 complete culture medium (90%1640 with 10% FBS) which contained the stimulator IL-10 (20 ng/ml, the final concentration) to culture in culture box (37℃, 5% CO_2). After 48h, total RNA of all samples was collected and lysed by TRIzol for qRT-PCR analysis subsequently. The detailed information of AIM2 ASO and Blimp1 siRNA target sequences is listed in Supplemental Table7.

RNA isolation and qRT-PCR

Total RNA was extracted using TRIzol reagent (Invitrogen). RNA quality control and concentration were detected by NanoDrop spectrophotometer (ND-2000, Thermo). mRNA was reverse-transcribed by the PrimeScript RT reagent Kit with a gDNA Eraser (TaKaRa, cat. RR047A) according to the manufacturer's protocols. qRT-PCR was carried out with TB Green Premix Ex Taq™ (Tli RNaseH Plus) (TaKaRa, cat. RR420A) by using thermocycler (Roche, LightCycler 96). The fold change of target gene expression was calculated as $2^{-\Delta\Delta Ct}$ ($\Delta\Delta Ct=\Delta Ct$ of experimental group – ΔCt of control group), which was normalized to the control group. mRNA primers for AIM2, Blimp-1, XBP1, Bcl-6, IRF4, MTA3, CD27, CD38 and GAPDH were purchased from Tsingke Biotechnology Co.. All gene primers are shown in Supplemental Table 8.

Pyrosequencing

Genome DNA of SLE patient $CD19^+$ B cells, healthy human $CD19^+$ B cells and healthy human $CD19^+$B cells stimulated with IL-10 were isolated using TIANamp Genomic DNA kit (TIANGEN, Cat. DP304-02). Approximately 20ng genome DNA was conversed by sulfite according to the manufacturer's protocols (Zymo research, EZ DNA Methylation™ kit, cat. D5002). Conversed genome DNA was used as amplification template. The targeted fragment from promoter of AIM2 was amplified using special primers and then was sequenced by Pyrophosphate sequencer (QIAGEN, PyroMark Q24). Primers for AIM2 were purchased from Tsingke Biotechnology Co.. Primer sequences are shown in Supplemental Table 9.

Histology

Mouse kidneys from pristane models were fixed by paraformaldehyde of which the concentration was 4% and then embedded in paraffin. Sliced sections of 5 μm thickness were affixed into HistoBond® adhesive microscopic slides. These slides were stained with H&E (hematoxylin and eosin) and then stored at room temperature. For the immunofluorescence (IF) assay, slides were incubated at 70°C for one hour to dissolve the wax. The slides were further dewaxed by followed sequential incubation: xylene for 10 minutes, 95% ethanol for 15 minutes, and 70% ethanol for 15 minutes. And then, the slides were incubated in blocking buffer (5% FBS in PBST) for 30 minutes at room temperature. Sections were incubated with primary and secondary antibodies at 4°C overnight and 30 minutes, respectively. The following antibodies were used: anti-C3 (Abcam, cat. ab11862), HRP-conjugated Affinipure Goat anti-rabbit IgG (H+L) (Proteintech, cat. SA00001-2) and Alexa Fluor® 488 anti-mouse IgG antibody (Abcam, ab150117). Immune-complex (IC) depositions in the mouse kidneys were analyzed and evaluated using a digital fluorescence microscope (Leica). We used NLS Elements Basic Research Imaging Software (Leica) to record and analyze fluorescent staining for presentation.

Multi-IHC staining

Slides of lupus skin lesions, healthy skin and tonsil tissues were deparaffinized by xylene, rehydrated, and washed in sterile water and then boiled in citrate buffer (Servicebio, cat. G1202-250ML, pH6.0) for antigen epitope retrieval. In multi-IHC experiments, firstly, protein blocking was performed using blocking dilution (PerkinElmer, ARD1001A) to reduce nonspecific binding. Secondly, primary antibodies were incubated for 1 hour at room temperature (primary antibodies including anti-CD4 (MXB Biotechnologies, RMA-0620), anti-human Bcl-6 (Abcam, cat. ab33901), anti-human CD19 (Abcam, cat. ab195896), and anti-human AIM2 (Abcam, cat. ab204995)). Next, incubation with secondary antibody (Opal polymer HRP Ms+Rb (PerkinElmer, ARH1001A)) was performed at room temperature for 10 minutes. And then TSA visualization was performed with the Opal seven-color IHC Kit (PerkinElmer, NEL801001KT) containing fluorophores DAPI, Opal 520, Opal 540, Opal 570, Opal 620, Opal 650, and Opal 690. Finally, the antibody-TSA complex was removed in citrate buffer (Servicebio, cat. G1202-250ML, pH6.0) by microwave treatment. Multi-IHC of tonsil tissue was conducted in the following order: CD19 (Opal 690), CD4 (Opal540), Bcl6 (Opal 520), and Aim2 (Opal620).

Multi-IHC of skin tissue was optimized by performing a duplex experiment (CD19, Opal 570 and Aim2, Opal 650). All TSA staining of tissues were finished with the fluorophores DAPI counterstain.

IHC Tissue imaging and analysis

We used the PerkinElmer Vectra multispectral imaging system (PerkinElmer, Vectra 3.0.3) to scan all slides. All multispectral images were deconvoluted using spectral libraries built from images of single stained tissues for each reagent by inForm Advanced Image Analysis software (PerkinElmer, inForm 2.3.1).

qRT-PCR for CD19^{+}B cell from from healthy human and SLE patients

We sorted CD19^{+} B cells from healthy human and SLE patients by magnetic beads (Miltenyi Biotec, 130-050-301). Total RNA were extracted by TRIzol reagent (Invitrogen), and then we got cDNA by RT(TaKaRa, cat. RR047A). qPCR was carried out with TB Green Premix Ex Taq^{TM} (Tli RNaseH Plus) (TaKaRa, cat. RR420A) by using thermocycler (Bio-Rad CFX). The fold change of target gene expression was calculated as $2^{-\Delta\Delta Ct}$ ($\Delta\Delta$Ct=ΔCt of experimental group – ΔCt of control group), which was normalized to the control group. mRNA primers for ASC, NLRP1, NLRP3, NLRP6, NLRP12, IFI16, NLRC4 and GAPDH were purchased from Tsingke Biotechnology Co.. Clinical sample information were listed in supplemental Table10. All gene primers are shown in supplemental Table11.

CD19^{+}B cell were stimulated by steroids Dexamethason(DEX) and qRT-PCR

We sorted CD19^{+}B cells from healthy human and SLE patients by magnetic beads (Miltenyi Biotec, 130-050-301). 2x10^{6} CD19^{+}B cells from healthy human were cultured by complete medium (90%1640 with 10%FBS) in 24 pore plate, and stimulated by Dexamethason with different concentration(Final concentration: 0μg/ml, 5μg/ml,10μg/ml). All sample were collected after 48h by stimulated by DEX. Total RNA were extracted by TRIzol reagent (Invitrogen), and then we got cDNA by RT(TaKaRa, cat. RR047A). qPCR was carried out with TB Green Premix Ex Taq^{TM} (Tli RNaseH Plus) (TaKaRa, cat. RR420A) by using thermocycler (Bio-Rad CFX). The fold change of target gene expression was calculated as $2^{-\Delta\Delta Ct}$ ($\Delta\Delta$Ct=ΔCt of experimental group – ΔCt of control group), which was normalized to the control group. mRNA primers for AIM2, ASC, NLRP1, NLRP3, NLRP6, NLRP12, IFI16, NLRC4 and GAPDH were purchased from Tsingke Biotechnology Co.. All gene primers are shown in supplemental Table11. Sample information from healthy donor was listed in supplemental Table12.

Confocal

We sorted CD19^{+} B cells from healthy human and SLE patients by magnetic beads (Miltenyi Biotec, 130-050-301). Firstly, 2x10^{6} CD19^{+}B cells were incubated by surface marker 1st antibody for 30min at 4℃ in the dark, and then washed with 1xPBS. Secondly, cells were incubated by surface marker 2nd antibody for 30min at 4℃ in the dark, and then washed with 1xPBS. Thirdly, for intracellular staining, cells were fixed and permeabilized with a Transcription Factor Buffer Set(BD, 562574), and then were respectively incubated with 1st and

2nd antibody at 4℃ in the dark. Finally, all sample were photoed by confocal microscopy (Germany, Zeiss LSM 780). All antibodies including CD27 (Abcam, ab131254), CD38 (Abcam, ab235118), IgD (Biolegend, 348205), AIM2(Abcam, ab204995, ab180665), AF594 Goat anti-Rabbit (Abcam, ab150080), AF488 Goat anti-Mouse (Abcam, ab150113).Sample information from healthy donor was listed in supplemental Table12.

CHIP-qRT-PCR

PBMCs of healthy volunteers and SLE patients were sorted by Ficoll-Paque Plus (GE healthcare, cat. 17-1440-03) , and $CD19^+$ B cells from PBMCs were sorted with magnetic beads (Miltenyi Biotec, 130-050-301). 1.5×10^6 $CD19^+$ B cells stimulated with IL-10 (20ng/ml) were collected after 72h. After cross-linking with 1% formaldehyde, stopping the cross-linking with glycine, and performing cell lysis to obtain nuclear lysate according to the kit instructions (Abcam, ab500). 1.5×10^6 cells/CHIP was incubated with antibodies at 4°C overnight, and the amount of H3AC (Active Motif, 61637), H3K4me3 (Active Motif, 61379), H3K9me3 (Active Motif, 61013) and H3K27me (Active Motif, 61017) antibody is 5μg/CHIP equally. The next day, after adding 40μl protein A agarose beads, rotating at 4°C for 1h. The magnetic bead-antibody-antigen complex was washed for 4 times using the 1×CHIP buffer in the kit to remove unbound proteins, and then de-crosslinked and digested with proteinase K. The magnetic bead-antibody-antigen was separated by heating at 98°C for 10 minutes, and centrifuged to obtain the DNA supernatant, and finally the DNA concentration was determined. The primers for different positions in the AIM2 promoter region were designed and specific identification was performed, and then the reaction was performed according to the qRT-PCR system and the relative quantitative value was calculated. Sample information from healthy donor and SLE patients were listed in supplemental Table13. Primers for AIM2 promoter CHIP-qRT-PCR were listed in supplemental Table14.

Human AIM2 promoter sequence

GTCAGTAACGGTGAGAGTTTTTCCCTTCTTGGGCCAGTTGCTACAGTAGGAGCCTCC
AGTCTTCTGCTTATGGCATAAACCTTGCTGTCAGTACTCTAGAAGTCAGGGAGGAAA
GGGCTTGGGGATCCCACTTTTCTCCGTGAGACTGTTAACAAACCTCCTTTTTCAGCAC
AGTTGCCTCCTTTCCCTTCTGTGTCTTGTGTCACCAAGTTGGGTATCTCTCTGATTAA
ATTTCTTGGTAAAACAAAGCTGGACGATGAGTACTTGTCTCTCTGCC**CCAGGTGCA**
GCAGTGTACCTGCTCCTCATTGAGACCACTTCCCAGCTTAATCACTTAACTCAC
CTTTTACAGGACCTGAAGCCTCAAATCAGTACGCCTCTCTGGGGTTGGTGCTTG
TTGGGTTCTTCCTCAACAGGGAGCTTTCTCTCGTTTACTCAGTAGGTTACCAAA
TTCATACACTTCACATCTTTTTAATTCTTACATATTGTTCTCTTTGGTGTTCTTT
CGCCTTGTGTGATCCACTCATTCATGCATTCACTCATTCAACGAATATTTTCTGAAC
ACCTTCTCTGTGCCTGGCACTGTGTCAGGTGAAG**TGGATGCAGTGAGCCAGTGGG**
CAGAAATCTCTTCCCTCATGTGCCTTACATTTTAGGAGCATGAGGCAGAGAATA
AACAAACAAATGACATATACCGTATGTCAGATGATGGTCAGTAACACGTGTGG
GAAAAGATAATGTGGGGAAGAGGTAGAGAACATGCTGGTCAGAGAAGCCCCAC
AGATAAGGTGCTATTTGAGCTGAAAGCTGGCAAAAGTGTAGAAACAAGCCTTGCAG
TAGAAAGTG**AAGGAGGTCCTGCAGCTCTGGGCTGATTACCTGGGCATGGTTAG**
GTGCTCTTCCTCTGCTCCTTGAGCATCTTGAGCATGCTTTAATTATAACACTTATCA
TCATGTCCTATTGTGATTATTAAATCACATGTCTGTTTTCATGACTT**GGCAGAGCCG**
CTCAACATCTGGAACTTTGTCTTACTAACTTTTATATTCTCAGAATCTAATACAT

CATATTAGATGTTCAGTGCATAATTGTTAATTCAATGAATAAGAAAGGAAAACC
TCAAGTAAATTGCTGACTAGAATCAAGCCCTCAAGCTCTGAATTTAGGGTTAGG
AGCCTGGGTTAGGAAGAAAGACTGTATATATGCTTTTGAGGCAAGCAGTCAGTGA
GGGAATGAGGCATGATTACTTGCAGGTGGCTACAAGCAATAAAAATGCGGTCAAAA
AGGTTAGCAGAGGGATT**GCCCCACTAGTAGATAGGAACTGTTACAACA**GCCTGTG
ACAATGAAGACAGATTGCAAACTTCCTACTTAGGCACAGATGAGAATGTAACACAG
AAGAAACTGGCCGCAACTGGTTAAAGCCAAGATGGTCGAAAACTTGGCTGACTGCT
GACCCTTAGCTTCATTATGCCCTTAATATCATAAAACTTCCATGGGGAAATTCCCACT
CCCATCATGCACCTGACGCCATGACAGTTCCGGATTAACCATCTTTAGTCAAGAAAA
GGGTGGCACCCCGATTCCGGGAATTGCCCGCCCATTTCCCAGAAAACCCCTCCCCTT
GATTACAGAGTACTCCATACCTTCATTGTGCTTATTCATATAGTACGTGAACCACATT
GCCTGTGACTCAAATCTTTGAGTGCCTGCACTCCTCCCTTGAGGGTGTCGTTGGTTTT
GCTCCACAATAAAATGTCTATACTTTCGCTTGGGTCTCATTTTCAGATTCTCTTATGC
TTTGAAGACAAGAACCTGCACTGCCCTACTGGCAACACTTTTAGATACCCTCTGAGA
TCAGTGTGCTTGTTTATACCAGAAAAATGTCATCACCAGGCCCCTTAGTCACAAAGT
GTATCAAGAAGTCATCTGCGGTCATTTTTTACAGTTTCTAGGCCACTTTCTGTTTCCA
ATCAGTGTAGTTCCTATGTGGCATGACTCAGATACAAGTTTCT

P1 F: CCAGGTGCAGCAGTGTACC
R： GAGTGGATCACACAAGGCGA （245bp）
P2 F: TGGATGCAGTGAGCCAGTGG
R： GTGGGGCTTCTCTGACCAGC （181bp）
P3 F: GGCAGAGCCGCTCAACATC
R: CCTAACCCAGGCTCCTAACCC (186bp)
P4 F CACAGAAGAAACTGGCCGC
R GGCAATGTGGTTCACGTACT (294bp)

Statistical analysis

We used the software GraphPad Prism 8.0. to perform statistical analysis. All data was presented as the mean ± SEM and assessed for normal distribution and similar variance between groups. Statistical significance of groups was assessed using a two-tailed unpaired Student's t-tests for comparisons between two groups and one-way analysis of variance (ANOVA) with relevant post hoc tests for multiple comparisons. We used the 2-tailed Mann-Whitney U test for statistical analysis, when the data were not normally distributed or displayed unequal variances between two groups. The correlation analysis of two indexes was performed using Pearson's r test or Spearman's r test (for abnormally distributed data). No statistical method was used to predetermine the sample size. All animals were allocated randomly to treatment groups.

Study approval

All samples studies from human were approved by the ethics committee of the Second Xiangya Hospital of Central South University. We obtained the written informed consent from all subjects. All animal care protocols and experiments were reviewed and approved by the Animal Care and

Use Committees of the Laboratory Animal Research Center at the Second Xiangya hospital of Central South University.

Supplementary Fig 1

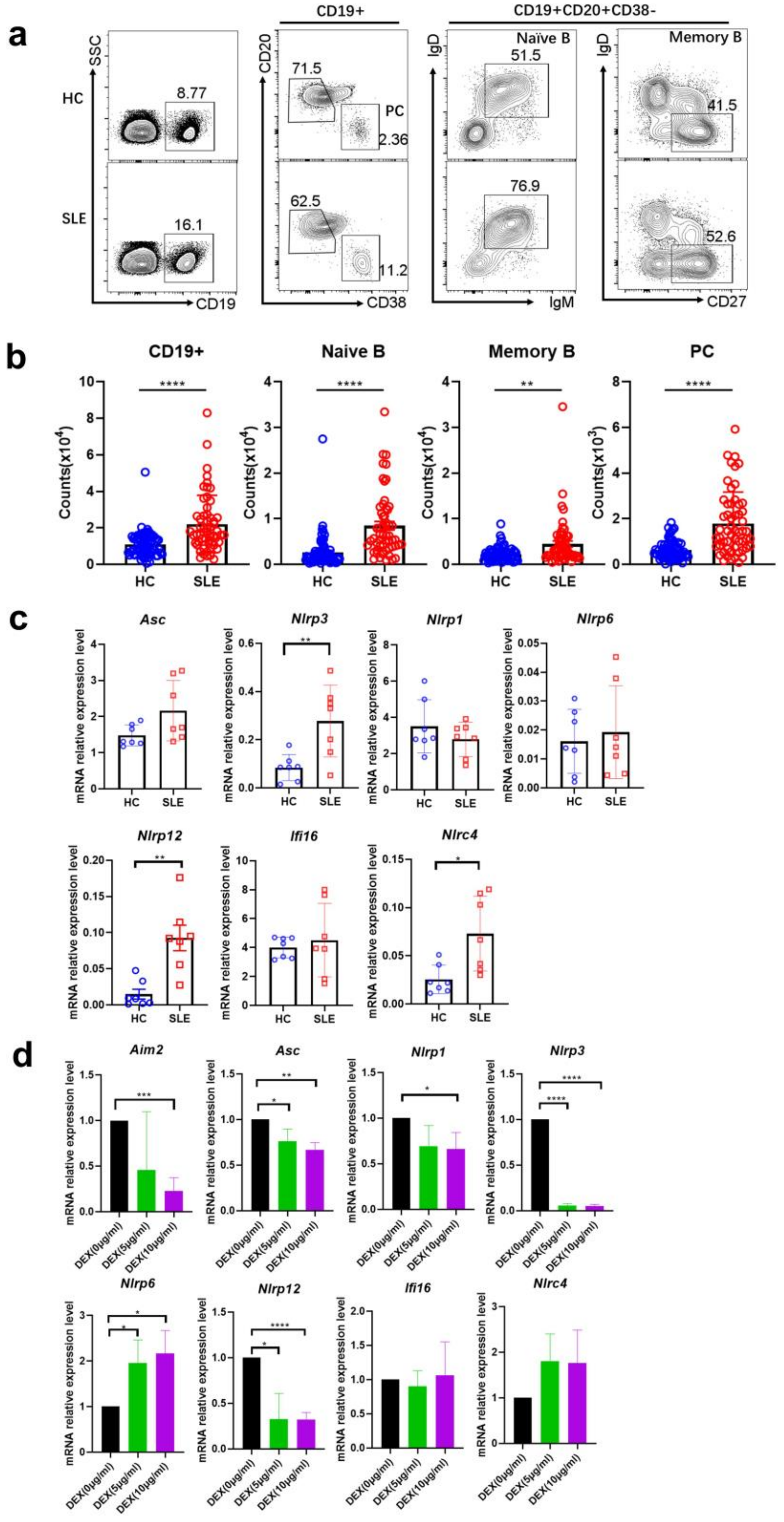

Supplementary Fig 1. Increased number of B cells and B cell subtypes in SLE circulation

a. The representative flow figure for plasma cell, naïve B cell and memory B cell gating.
b. The statistical analysis of numbers of plasma cells, naïve B cells and memory B cells from healthy controls (HCs) (n = 70), SLE patients (n = 56).
c. The statistical analysis of mRNA expression in B cells from HCs (n = 7), SLE patients (n = 7).
d. The statistical analysis of mRNA expression in B cells treated by Dexamethasone (Dex) (n = 3). Horizontal bars represent the mean ± SEM. * $p < 0.05$. ** $p < 0.01$, *** $p < 0.005$, **** $p < 0.0001$.

Supplementary Fig 2

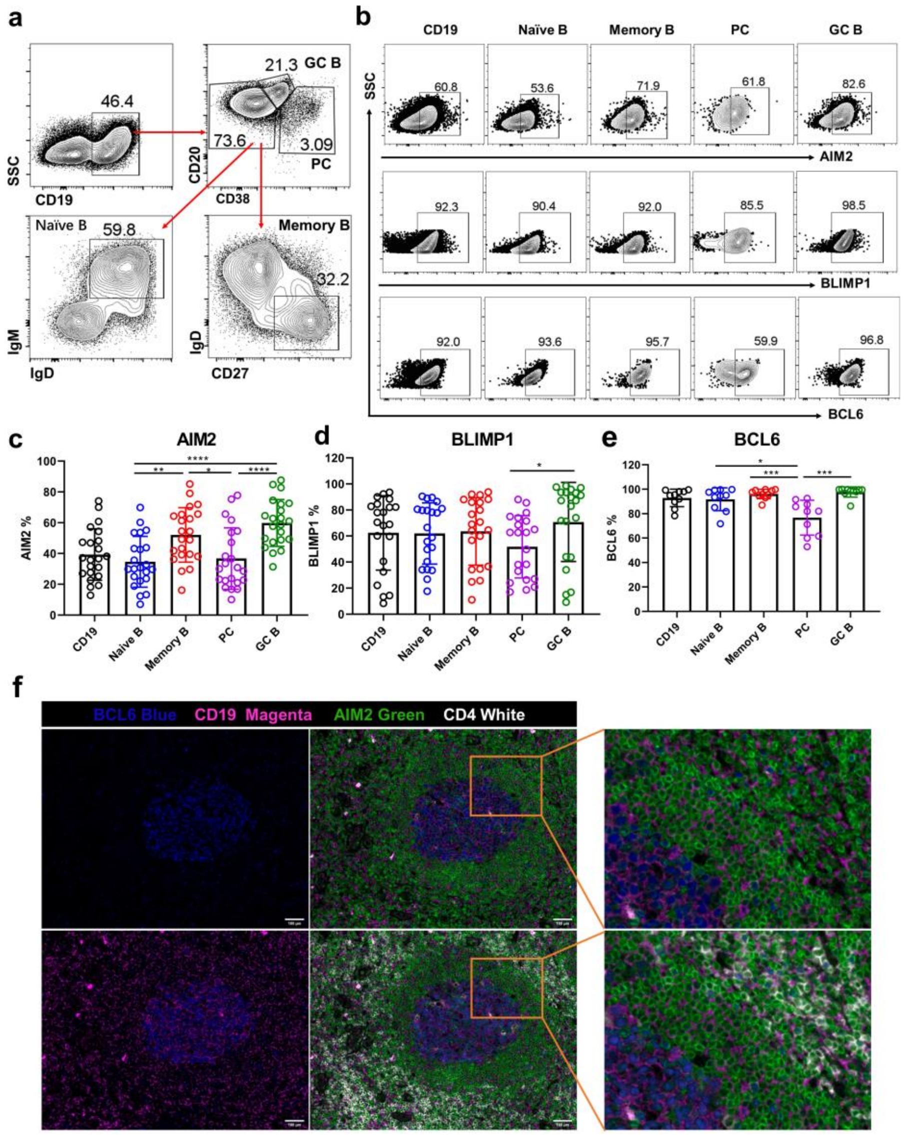

Supplementary Fig 2. The increased AIM2 expression in human tonsil B cells

The expression of AIM2 was detected by flow cytometry and multicolor microscopy.
a. Representative flow diagram of gating for GC B cells, plasma cells, naïve B cells, and memory B cells.
b. Representative flow cytometry analysis of AIM2, Blimp-1 and Bcl-6 expression in GC B cells, plasma cells, naïve B cells, and memory B cells.
c-e. Statistical analysis of $AIM2^+$, Blimp-1^+, and Bcl-6^+ cells in the populations of GC B cells, plasma cells, naïve B cells, and memory B cells.
f. The location and expression levels of AIM2 in tonsil samples. The follicles are represented by Bcl-6 in blue, $CD19^+$ B cells are surrounded by $CD4^+$ T cells in white and $AIM2^+$ cells are labeled in green. Horizontal bars represent the mean ± SEM. * $p < 0.05$. ** $p < 0.01$, *** $p < 0.005$, **** $p < 0.0001$.

Supplementary Fig 3

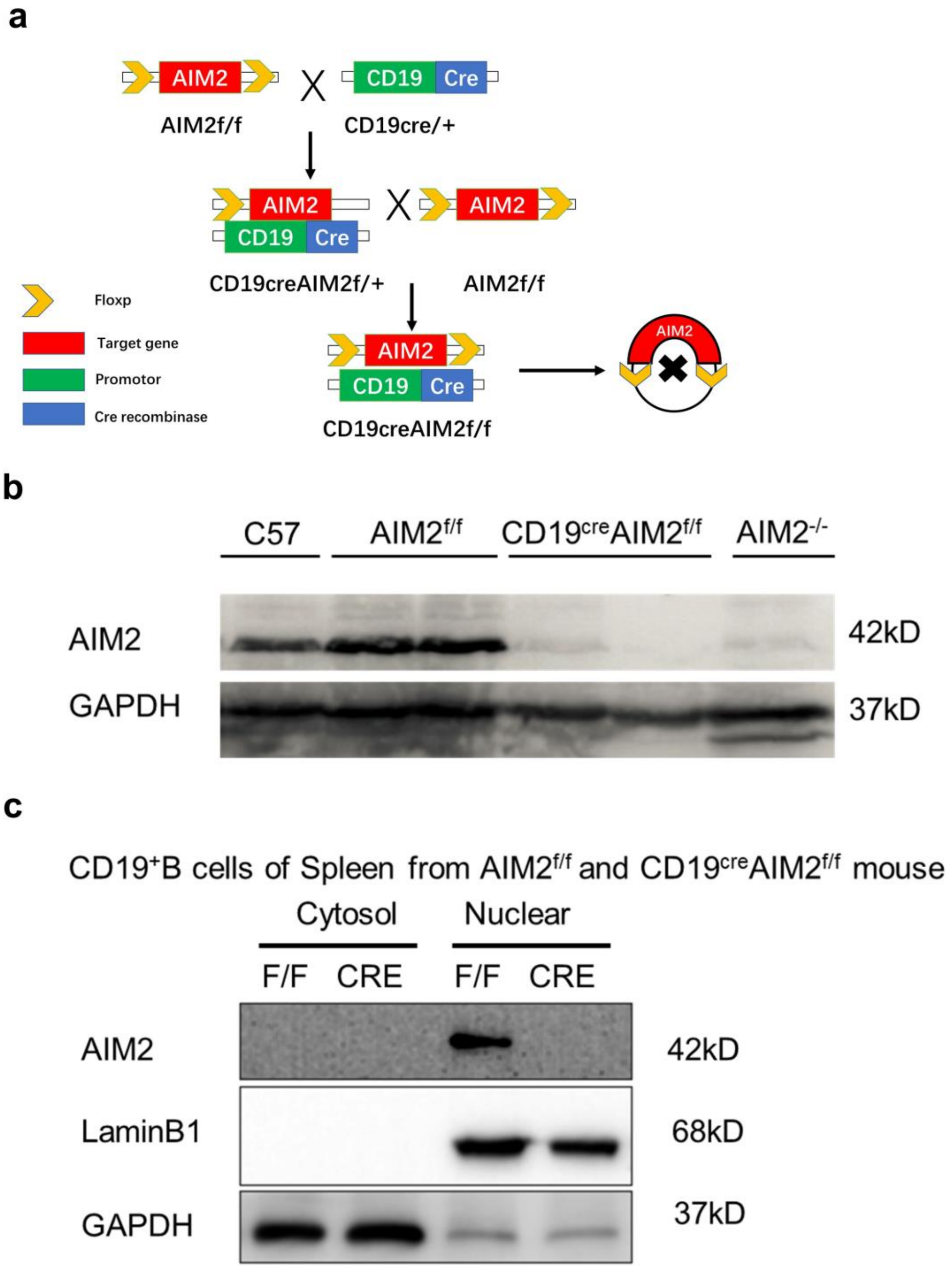

Supplementary Fig 3. Generation of CD19^{cre}AIM2$^{f/f}$ mice

a. The Cre-loxP system used for CD19^{cre}AIM2$^{f/f}$ mouse generation.

b. Confirmation of the AIM2 knockout effect in CD19^{cre}AIM2$^{f/f}$ mice by western blotting.

c. The localization of AIM2 in B cells, as determined by western blotting.

Supplementary Fig 4

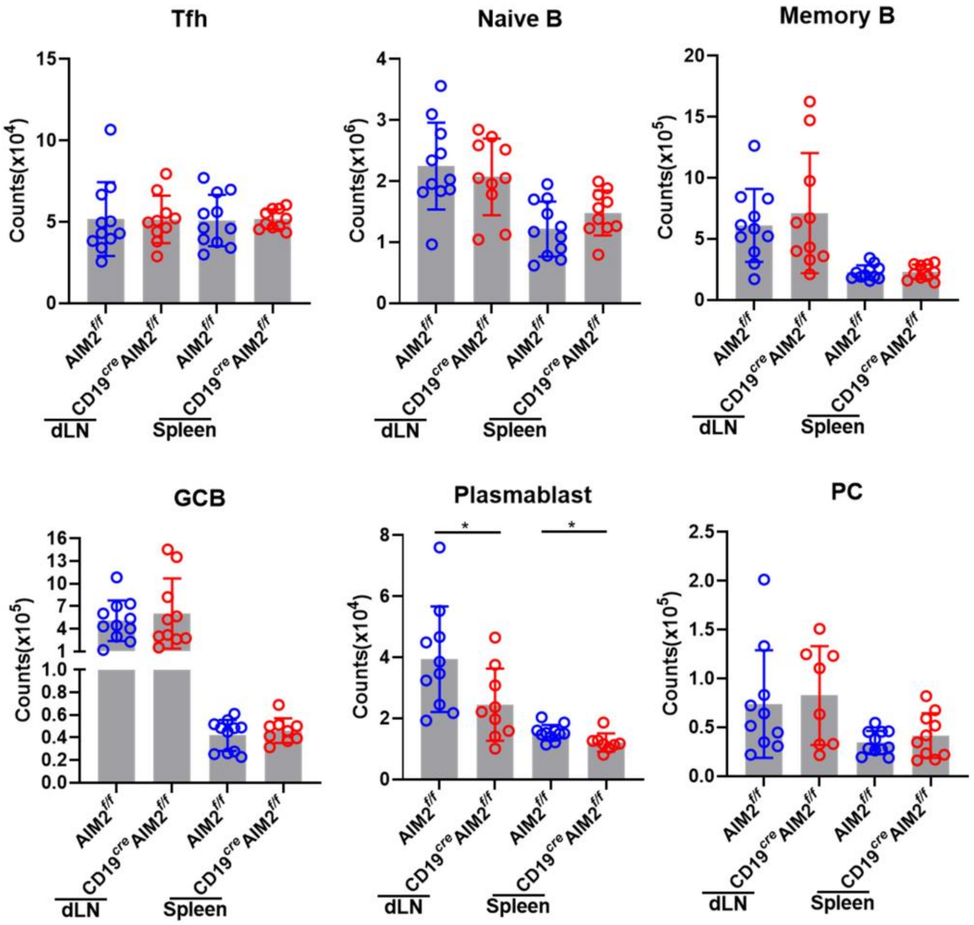

Supplementary Fig 4. The statistical analysis of numbers of Tfh cells, naïve B cells, memory B cells, GC B cells, plasmablast cells and plasma cells in dLNs and spleens of $CD19^{cre}AIM2^{f/f}$ mice and $AIM2^{f/f}$ mice. Horizontal bars represent the mean ± SEM. * $p < 0.05$. ** $p < 0.01$, *** $p < 0.005$, **** $p < 0.0001$.

Supplementary Fig 5

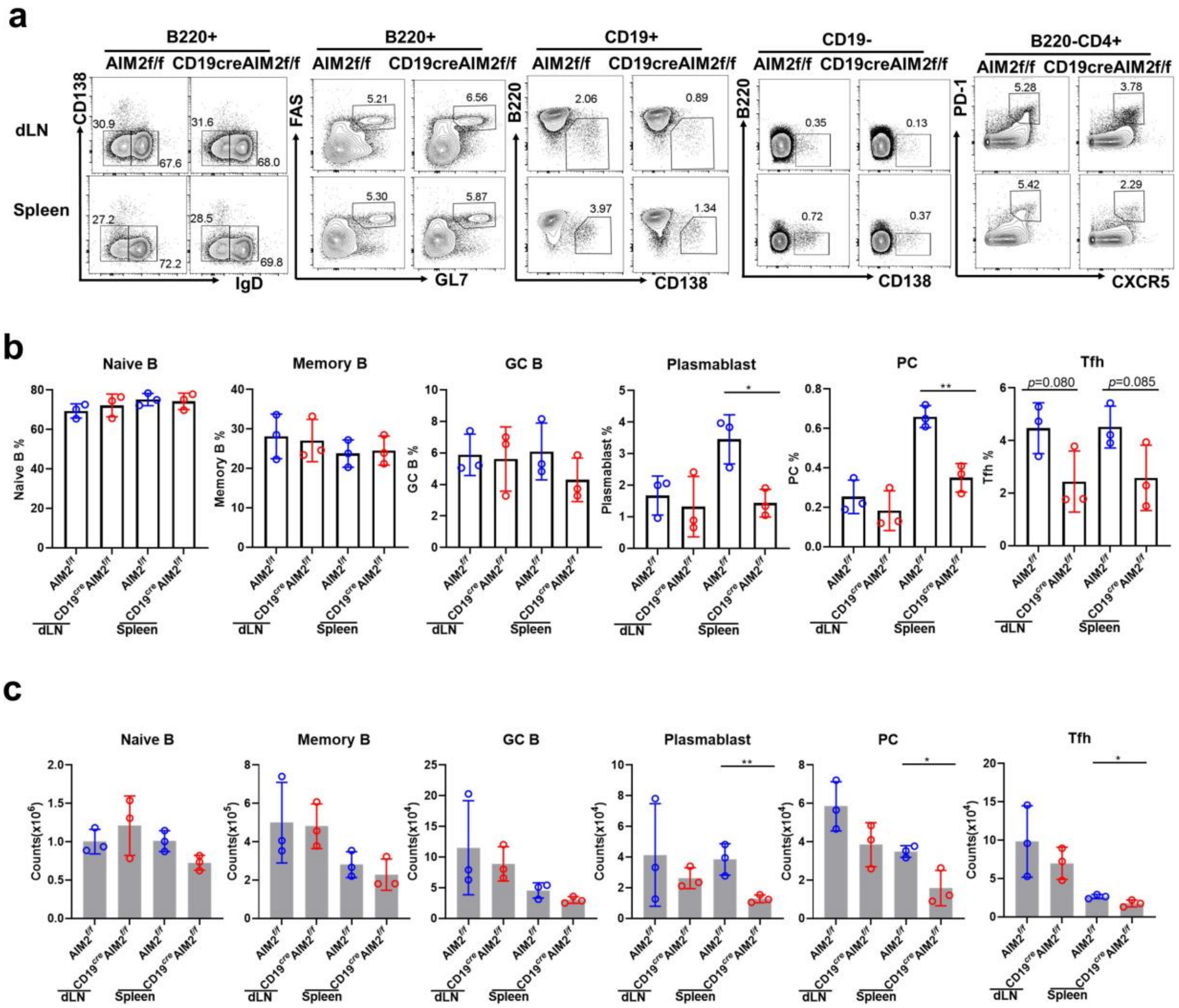

Supplementary Fig 5. The KLH-induced response in CD19creAIM2f/f mice on Day 28

a. On Day 28 after KLH immunization, cells were collected from the dLNs and spleens of the CD19creAIM2f/f mice and AIM2f/f mice. Representative flow cytometry diagrams and statistical analysis of the percentages of naïve B cells, memory B cells, GC B cells, plasmablast cells, plasma cells and Tfh cells in the dLNs and spleens of the CD19creAIM2f/f mice and AIM2f/fmice.

b. The statistical analysis of percentage of naïve B cells, memory B cells, GC B cells, plasmablast cells, plasma cells and Tfh cells in dLNs and spleens of CD19creAIM2f/f mice and AIM2f/f mice.

c. The statistical analysis of numbers of naïve B cells, memory B cells, GC B cells, plasmablast cells, plasma cells and Tfh cells in dLNs and spleens from CD19creAIM2f/f mice and AIM2f/f mice. Horizontal bars represent the mean ± SEM. * $p < 0.05$. ** $p < 0.01$, *** $p < 0.005$, **** $p < 0.0001$.

Supplementary Fig 6

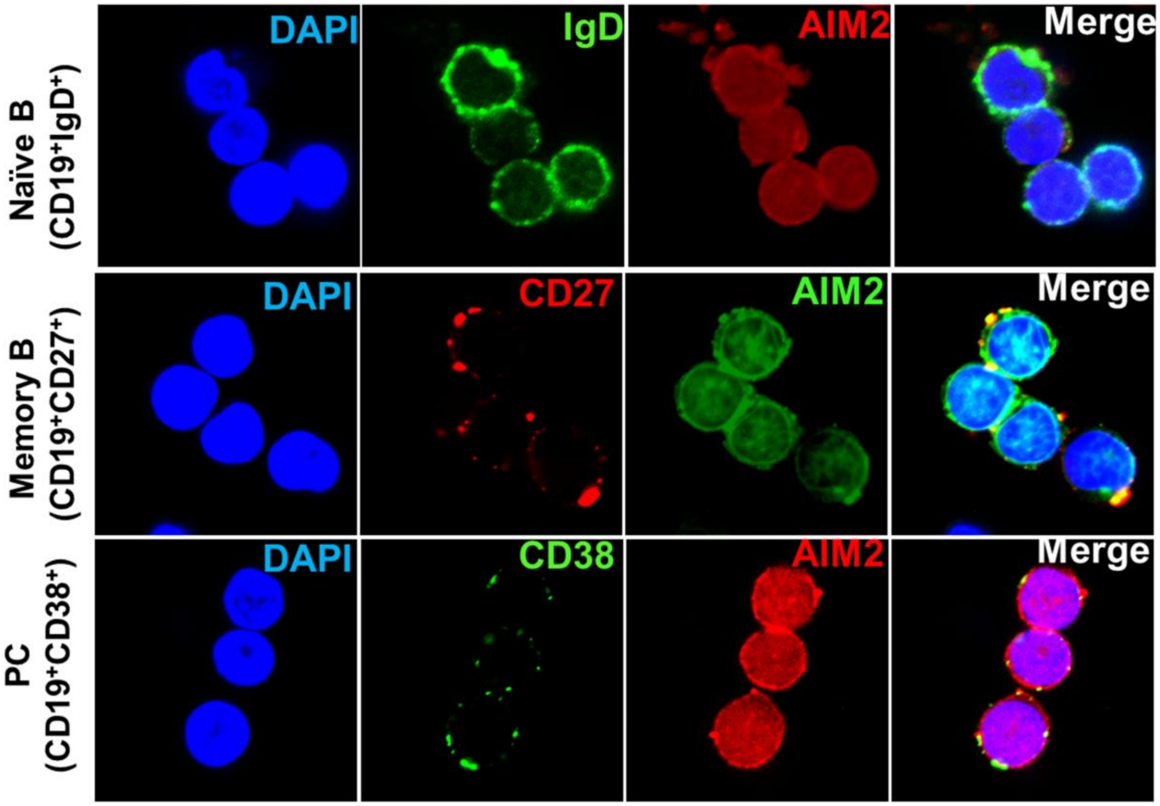

Supplementary Fig 6. The location of AIM2 in different B cell subtypes
AIM2 expression in NC peripheral naïve B cells ($CD19^{+}IgD^{+}$), memory B cells ($CD19^{+}CD27^{+}$), and plasma cells ($CD19^{+}CD38^{+}$) was observed by confocal microscopy.

Supplementary Fig 7

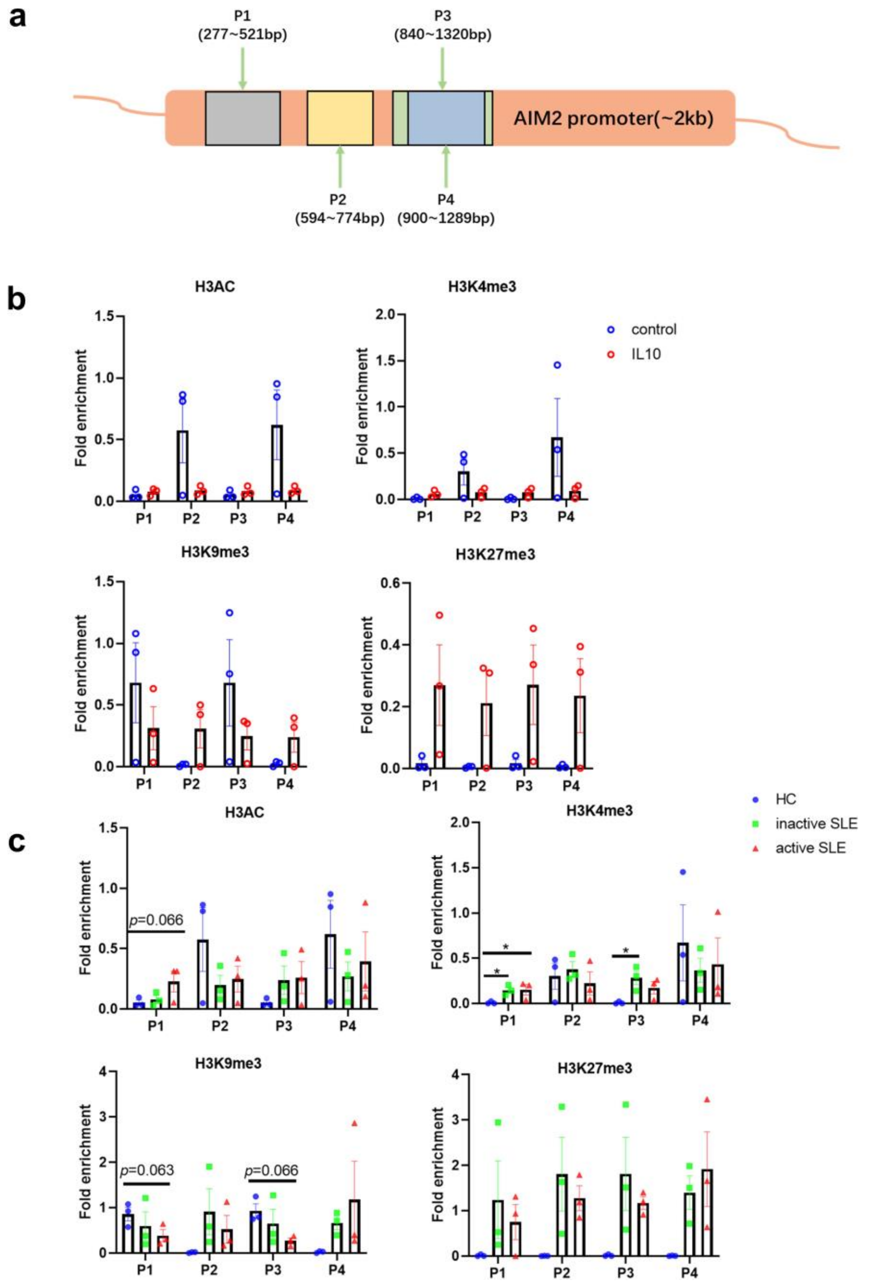

Supplementary Fig 7. The detection of histone modification on AIM2 promoter region

a. The region of position to detect the enrichment of histone modifications.

b. The enrichment of histone modifications on AIM2 promoter region after IL-10 treatment (n=3).

c. The enrichment of histone modifications on AIM2 promoter region in HC (n =3), inactive SLE (n =3) and active SLE patients (n =3). Horizontal bars represent the mean ± SEM. * $p < 0.05$. ** $p < 0.01$, *** $p < 0.005$, **** $p < 0.0001$.

Table S1.

Gender	Age	Clinical diagnosis
F	15	Snoring/Hypertrophy of tonsils/Chronic tonsillitis
M	7	Snoring/Hypertrophy of tonsils/Adenoidal hypertrophy
M	5	Snoring/Hypertrophy of tonsils/Adenoidal hypertrophy
M	6	Hypertrophy of tonsils/Adenoidal hypertrophy/Chronic tonsillitis
F	26	Snoring/Hypertrophy of tonsils/Chronic tonsillitis
F	4	Hypertrophy of tonsils/Adenoidal hypertrophy/Chronic tonsillitis
F	7	Hypertrophy of tonsils/Adenoidal hypertrophy/Chronic tonsillitis
M	6	Snoring/Hypertrophy of tonsils/Adenoidal hypertrophy
M	9	Hypertrophy of tonsils/Adenoidal hypertrophy
M	5	Hypertrophy of tonsils/Adenoidal hypertrophy/Chronic tonsillitis
M	8	Hypertrophy of tonsils/Adenoidal hypertrophy/Chronic tonsillitis
F	19	Hypertrophy of tonsils/Chronic tonsillitis
M	13	Snoring/Hypertrophy of tonsils/Adenoidal hypertrophy
F	12	Snoring/Hypertrophy of tonsils/Adenoidal hypertrophy
M	7	Hypertrophy of tonsils/Adenoidal hypertrophy
F	13	Snoring/Hypertrophy of tonsils/Chronic tonsillitis
F	16	Snoring/Hypertrophy of tonsils/Adenoidal hypertrophy
F	9	Snoring/Hypertrophy of tonsils/Adenoidal hypertrophy
F	16	Snoring/Hypertrophy of tonsils/Adenoidal hypertrophy
M	13	Snoring/Hypertrophy of tonsils/Adenoidal hypertrophy

F	8	Snoring/Hypertrophy of tonsils/Adenoidal hypertrophy
F	10	Snoring/Hypertrophy of tonsils/Adenoidal hypertrophy

Table S1. Clinical sample information of human tonsil tissues from Department of Otolaryngology head and neck surgery of the Second Xiangya hospital of Central South University for Flow Cytometry analysis.

Table S2.

NO.	Gender	Age (Years)	SLEDAI SCORE	NO.	Gender	Age (Years)
SLE1	F	53	0	HC8	F	24
SLE2	F	46	2	HC9	F	23
SLE3	F	49	0	HC10	F	23
SLE4	F	18	8	HC11	F	39
SLE5	F	53	4	HC12	F	37
SLE6	F	51	3	HC13	F	33
SLE7	F	25	2	HC14	F	33
SLE8	F	49	5	HC15	F	33
SLE9	F	33	5	HC16	F	24
SLE10	F	33	8	HC17	F	36
SLE11	F	35	8	HC18	F	30
SLE12	F	13	3	HC19	F	32
SLE13	F	51	8	HC20	F	49
SLE14	F	32	4	HC21	F	32
SLE15	F	33	6	HC22	F	55
SLE16	F	27	4	HC23	F	31
SLE17	F	46	8	HC24	F	60
SLE18	F	45	2	HC25	F	48
SLE19	F	31	6	HC26	F	31

SLE20	F	25	12	HC27	F	32
SLE21	F	28	10	HC28	F	37
SLE22	F	23	6	HC29	F	31
SLE23	F	19	16	HC30	F	52
SLE24	F	54	5	HC31	F	34
SLE25	F	49	0	HC32	F	33
SLE26	F	39	2	HC33	F	57
SLE27	F	17	6	HC34	F	50
SLE28	F	49	0	HC35	F	31
SLE29	F	56	2	HC36	F	25
SLE30	M	44	8	HC37	F	33
SLE31	F	37	7	HC38	F	40
SLE32	F	25	4	HC39	F	37
SLE33	F	48	2	HC40	F	36
SLE34	F	37	2	HC41	F	50
SLE35	F	76	0	HC42	F	30
SLE36	F	66	2	HC43	F	60
SLE37	F	17	4	HC44	F	31
SLE38	F	30	4	HC45	F	30
SLE39	F	12	6	HC46	F	30
SLE40	F	28	4	HC47	F	54
SLE41	F	25	0	HC48	F	20

SLE42	F	39	2	HC49	F	35
SLE43	F	28	8	HC50	F	69
SLE44	F	49	8	HC51	F	52
SLE45	F	31	0	HC52	F	27
SLE46	F	35	8	HC53	F	31
SLE47	F	38	6	HC54	F	51
SLE48	F	41	18	HC55	F	56
SLE49	F	43	2	HC56	F	28
SLE50	F	57	0	HC57	F	60
SLE51	F	39	6	HC58	F	45
SLE52	F	28	8	HC59	F	20
SLE53	F	31	6	HC60	F	35
SLE54	F	53	2	HC61	F	44
SLE55	F	16	8	HC62	F	30
SLE56	F	12	4	HC63	F	66
HC1	F	25		HC64	F	40
HC2	F	26		HC65	F	34
HC3	F	24		HC66	F	30
HC4	F	25		HC67	F	20
HC5	F	26		HC68	F	50
HC6	F	27		HC69	F	37
HC7	F	24		HC70	F	30

Table S2. Clinical sample information of Systemic Lupus Erythematosus (SLE) patients from Department of dermatology of the Second Xiangya hospital of Central South University for Flow Cytometry analysis (Inactive SLE: SLEDAI SCORE≤4, Active SLE: SLEDAI SCORE>4), and healthy controls (HCs) from the Physical Examination Center of the Second Xiangya hospital of Central South University for Flow Cytometry analysis.

Table S3.

NO.	Gender	Age (Years)	SLEDAI SCORE	NO.	Gender	Age (Years)
SLE1	F	52	4	HC1	F	30
SLE2	F	44	6	HC2	F	29
SLE3	F	42	2	HC3	F	26
SLE4	F	34	18	HC4	F	24
SLE5	F	40	6	HC5	F	25
SLE6	F	24	6	HC6	F	31
SLE7	F	47	4	HC7	F	35
SLE8	F	25	4	HC8	F	37
SLE9	F	43	0	HC9	F	28
SLE10	F	43	0	HC10	F	21
SLE11	F	38	0	HC11	F	32
SLE12	F	23	4	HC12	F	35
SLE13	F	48	4	HC13	F	20
SLE14	F	32	6			
SLE15	F	24	8			
SLE16	F	23	4			
SLE17	F	17	6			

Table S3. Clinical sample information of SLE patients from Department of dermatology of the Second Xiangya hospital of Central South University (Inactive SLE: SLEDAI SCORE≤4, Active SLE: SLEDAI SCORE>4), and HCs from the Physical Examination Center of the Second Xiangya hospital of Central South University for pyrosequencing.

Table S4.

**Species**	**Gene**	**Sequence (5’→3’)**
Human	Aim2	Former: TCAAGCTGAAATGAGTCCTGC
		Reverse: CTTGGGTCTCAAACGTGAAGG
Human	Prdm1	Former: TAAAGCAACCGAGCACTGAGA
		Reverse: ACGGTAGAGGTCCTTTCCTTTG
Human	Bcl6	Former: ACACATCTCGGCTCAATTTGC
		Reverse: AGTGTCCACAACATGCTCCAT
Human	Pax5	Former: ACTTGCTCATCAAGGTGTCAG
		Reverse: TCCTCCAATTACCCCAGGCTT
Human	Xbp1	Former: CCCTCCAGAACATCTCCCCAT
		Reverse: ACATGACTGGGTCCAAGTTGT
Human	Mta3	Former: GACTTGACCGATAAGCAGAAACA
		Reverse: AGGGCAACACTGCACTTTCC
Human	Irf4	Former: GCTGATCGACCAGATCGACAG
		Reverse: CGGTTGTAGTCCTGCTTGC
Human	Cd27	Former: CAGAGAGGCACTACTGGGCT
		Reverse: CGGTATGCAAGGATCACACTG
Human	Cd38	Former: AGACTGCCAAAGTGTATGGGA
		Reverse: GCAAGGTACGGTCTGAGTTCC
Human	Gapdh	Former: GGAGCGAGATCCCTCCAAAAT
		Reverse: GGCTGTTGTCATACTTCTCATGG

Table S4. Primers for qRT-PCR

Table S5.

**Human AIM2: Sequence (5’→3’)**
Amplification primer
Former: GATTGGAAATAGAAAGTGGTTTAGAAA
Reverse: Biotin-CAAAAACCTACACTACCCTACTAACAA
Sequencing primer
S1: TGGTTTAGAAATTGTAAAAAATG

Table S5. Primers for pyrosequencing

Table S6.

Genes	Target sequence (5’→3’)
AIM2 ASO	TAAATAGCGCCTCACGTGTG
PRDM1 siRNA-1	GCAACTGGATGCGCTATGT

Table S6. Genes ASO and siRNA target sequences

Table S7.

NO.	Gender	Age (Years)
1	F	24
2	M	24
3	M	25
4	M	24
5	M	32
6	F	22
7	M	25
8	M	23
9	M	24
10	M	24
11	F	25
12	F	22
13	M	22
14	F	22
15	F	21

Table S7. Sample information of $CD19^+$ B cells of peripheral blood mononuclear cells (PBMCs) from healthy donors which were stimulated with IL-10.

Table S8.

**NO.**	**Gender**	**Age (Years)**	**SLEDAI SCORE**
DLE1	F	45	-
DLE2	M	45	-
DLE3	M	52	-
SLE1	F	28	25
SLE2	F	44	14
SLE3	F	24	8

Table S8. Clinical sample information of skin lesions for AIM2 immunohistochemistry (IHC) analysis in $CD19^+$ B cells.

Table S9.

NO.	Gender	Age (Years)	SLEDAI SCORE
SLE1	M	16	22
SLE2	F	46	20
SLE3	F	73	18
SLE4	F	19	16
SLE5	F	25	16
SLE6	F	27	16
SLE7	F	52	22
SLE8	F	40	16
SLE9	F	51	16
SLE10	F	27	16
SLE11	F	24	16
SLE12	F	23	6
SLE13	F	26	18
SLE14	F	15	4
SLE15	F	51	16
SLE16	F	26	16
SLE17	F	47	4
SLE18	F	30	8
SLE19	F	33	6
SLE20	F	52	26

SLE21	F	52	22
SLE22	F	49	16
SLE23	F	24	26
SLE24	F	24	21
SLE25	F	21	4
SLE26	F	24	16
SLE27	F	27	19
SLE28	F	41	25
SLE29	F	53	16
SLE30	F	19	4
SLE31	F	44	2
SLE32	F	56	19
SLE33	F	26	4
SLE34	F	19	18
SLE35	F	41	18
SLE36	F	20	6
SLE37	F	14	23
SLE38	F	26	18
SLE39	F	18	6
SLE40	F	27	22
SLE41	F	20	16
SLE42	F	33	21

SLE43	F	20	18
SLE44	F	35	27
SLE45	F	30	16
SLE46	F	30	20
SLE47	F	15	16
SLE48	F	28	16
SLE49	F	29	20
SLE50	F	27	6
SLE51	F	24	18
SLE52	F	23	18
SLE53	F	43	16
SLE54	F	53	8
SLE55	F	48	20
SLE56	F	46	18
HC1	F	50	-
HC2	F	42	-
HC3	F	34	-
HC4	F	26	-
HC5	F	29	-
HC6	F	28	-
HC7	F	34	-
HC8	F	35	-

HC9	F	40	-
HC10	F	33	-
HC11	F	37	-
HC12	F	37	-
HC13	F	49	-
HC14	F	41	-
HC15	F	23	-
HC16	F	47	-
HC17	F	39	-
HC18	F	32	-
HC19	F	25	-
HC20	F	29	-
HC21	F	41	-
HC22	F	44	-
HC23	F	42	-
HC24	F	39	-

Table S9. Clinical sample information of serum IL-10 ELISA analysis in SLE patients and HCs.

Table S10.

**NO.**	**Gender**	**Age (Years)**
HC1	F	21
HC2	M	25
HC3	M	24
HC4	F	26
HC5	F	23
HC6	F	24
HC7	F	24
SLE1	F	33
SLE2	M	32
SLE3	F	40
SLE4	F	21
SLE5	F	27
SLE6	F	31
SLE7	F	22

Table S10. qRT-PCR sample information of $CD19^+$ B cells of PBMCs from healthy donors and SLE patients.

Table S11.

Species	Gene	Sequence (5’→3’)
Human	Aim2	Former: TCAAGCTGAAATGAGTCCTGC
		Reverse: CTTGGGTCTCAAACGTGAAGG
Human	Asc	Former: TGGATGCTCTGTACGGGAAG
		Reverse: CCAGGCTGGTGTGAAACTGAA
Human	Nlrp1	Former: GCAGTGCTAATGCCCTGGAT
		Reverse: GAGCTTGGTAGAGGAGTGAGG
Human	Nlrp3	Former: GATCTTCGCTGCGATCAACAG
		Reverse: CGTGCATTATCTGAACCCCAC
Human	Nlrp6	Former: CCTACCAGTTCATCGACCAGA
		Reverse: CTCAGCAGTCCGAAGAGGAA
Human	Nlrp12	Former: ACCAGACCTTGACCGACCTT
		Reverse: GAGGACTCGGAGTTTGCAGC
Human	Ifi16	Former: GTTTGCCGCAATGGGTTCC
		Reverse: ATCTCCATGTTTCGGTCAGCA
Human	Nlrc4	Former: TGCCCAGAAATCGAAGCCC
		Reverse:GGCACCAAACTGCCGTATG
Human	Gapdh	Former: GGAGCGAGATCCCTCCAAAAT
		Reverse: GGCTGTTGTCATACTTCTCATGG

Table S11. Inflammasome gene primers for qRT-PCR

Table S12.

**NO.**	**Gender**	**Age (Years)**
HC1	M	21
HC2	F	26
HC3	F	25

Table S12. Healthy donor sample information for Dexamethason stimulation

Table S13.

NO.	Gender	Age (Years)
HC1	F	25
HC2	M	28
HC3	M	26
Inactive SLE1	F	35
Inactive SLE2	F	22
Inactive SLE3	F	31
Active SLE1	F	45
Active SLE2	F	45
Active SLE3	F	21

Table S13. CHIP-qRT-PCR sample information of $CD19^+$ B cells of PBMCs from healthy donors and SLE patients.

Table S14.

**Gene Fragment**	**Sequence (5'→3')**	**Length**
P1	Former: CCAGGTGCAGCAGTGTACC	245bp
	Reverse: GAGTGGATCACACAAGGCGA	
P2	Former: TGGATGCAGTGAGCCAGTGG	181bp
	Reverse: GTGGGGCTTCTCTGACCAGC	
P3	Former: GGCAGAGCCGCTCAACATC	186bp
	Reverse: CCTAACCCAGGCTCCTAACCC	
P4	Former: CACAGAAGAAACTGGCCGC	294bp
	Reverse: GGCAATGTGGTTCACGTACT	

Table S14**.** Primers for AIM2 promoter Chip-qRT-PCR
